# Comparison of computational methods for the identification of topologically associating domains

**DOI:** 10.1186/s13059-018-1596-9

**Published:** 2018-12-10

**Authors:** Marie Zufferey, Daniele Tavernari, Elisa Oricchio, Giovanni Ciriello

**Affiliations:** 1 Department of Computational Biology, University of Lausanne (UNIL), Lausanne, Switzerland; 2 Swiss Institute of Bioinformatics, Lausanne, Switzerland; 3 Swiss Institute for Experimental Cancer Research (ISREC), School of Life Sciences, École Polytechnique Fédérale de Lausanne (EPFL), Lausanne, Switzerland

**Keywords:** Topologically associating domain, Hi-C, Method comparison

## Abstract

**Background:**

Chromatin folding gives rise to structural elements among which are clusters of densely interacting DNA regions termed topologically associating domains (TADs). TADs have been characterized across multiple species, tissue types, and differentiation stages, sometimes in association with regulation of biological functions. The reliability and reproducibility of these findings are intrinsically related with the correct identification of these domains from high-throughput chromatin conformation capture (Hi-C) experiments.

**Results:**

Here, we test and compare 22 computational methods to identify TADs across 20 different conditions. We find that TAD sizes and numbers vary significantly among callers and data resolutions, challenging the definition of an average TAD size, but strengthening the hypothesis that TADs are hierarchically organized domains, rather than disjoint structural elements. Performances of these methods differ based on data resolution and normalization strategy, but a core set of TAD callers consistently retrieve reproducible domains, even at low sequencing depths, that are enriched for TAD-associated biological features.

**Conclusions:**

This study provides a reference for the analysis of chromatin domains from Hi-C experiments and useful guidelines for choosing a suitable approach based on the experimental design, available data, and biological question of interest.

**Electronic supplementary material:**

The online version of this article (10.1186/s13059-018-1596-9) contains supplementary material, which is available to authorized users.

## Background

The recent advent of chromatin conformation capture technologies has made it possible to systematically investigate spatial interactions between genomic loci at unprecedented resolution [1]. In particular, high-throughput sequencing of genome-wide interactions (Hi-C) has led to the identification and characterization of multiple structural elements composing the chromatin architecture ranging from DNA loops between loci less than a megabase apart [2, 3] to hubs of inter-chromosomal contacts [4] and chromosomal compartments [5]. Within this compartmentalization of the genome, topologically associating domains (TADs) have been described as chromatin regions more frequently interacting within themselves than among each other [6–8]. TADs have been reported as highly conserved across species [6] and cell types [9] and with a size ranging between 100 kb and 5 Mb [10]. Importantly, the functional relevance of these domains has been investigated in development [11, 12] and cell differentiation [13], as well as in disease contexts, where modifications of TAD boundaries (start and end points of the domain) have been associated with both genetic diseases [14] and cancer [15, 16].

These results prompted a great interest in TADs and, thus, in developing computational methods to identify such domains from Hi-C experiments. In the past few years, dozens of computational tools have been proposed and applied to multiple datasets. Initial assessments of a subset of these methods have already highlighted important differences between their output [17, 18]; however, a comprehensive comparison of many tools in terms of robustness to data resolution and normalization and method parameters, of concordance between the retrieved TAD sets, and of their ability to recapitulate TAD-associated biological features is missing. The underlying assumption of all these tools is that chromatin interactions are greater within TADs than among them and the distribution of contacts towards upstream or downstream chromosomal regions is mostly skewed at TAD boundaries. To quantify these trends and identify such boundaries, most of the initial approaches relied on fractioning each chromosome into small fixed-size genomic intervals (or *bins*) and defining a linear score associated to each bin [2, 6, 19–28]. More recently, alternative approaches have been designed relying either on statistical models of the interaction distributions [29–32] or on clustering approaches applied to the matrix of chromatin contacts [33–35] or on concepts borrowed from graph theory, such as network modularity, where the Hi-C contact matrix is thought of as the adjacency matrix of a graph with bins as nodes and TADs as dense subnetworks or “communities” within such graph [36–38]. Ultimately, these tools return a partition of the chromosome into disjoint or overlapping domains, the latter potentially organized in a hierarchy of nested domains. Given the high number of proposed tools and the relevance of these domains in regulating chromatin functions, questions arise on how much these methods depend on the quality of the data, whether they are interchangeable, and whether TADs identified by these callers are associated with key biological features characteristic of chromatin domains.

**Fig. 1:**
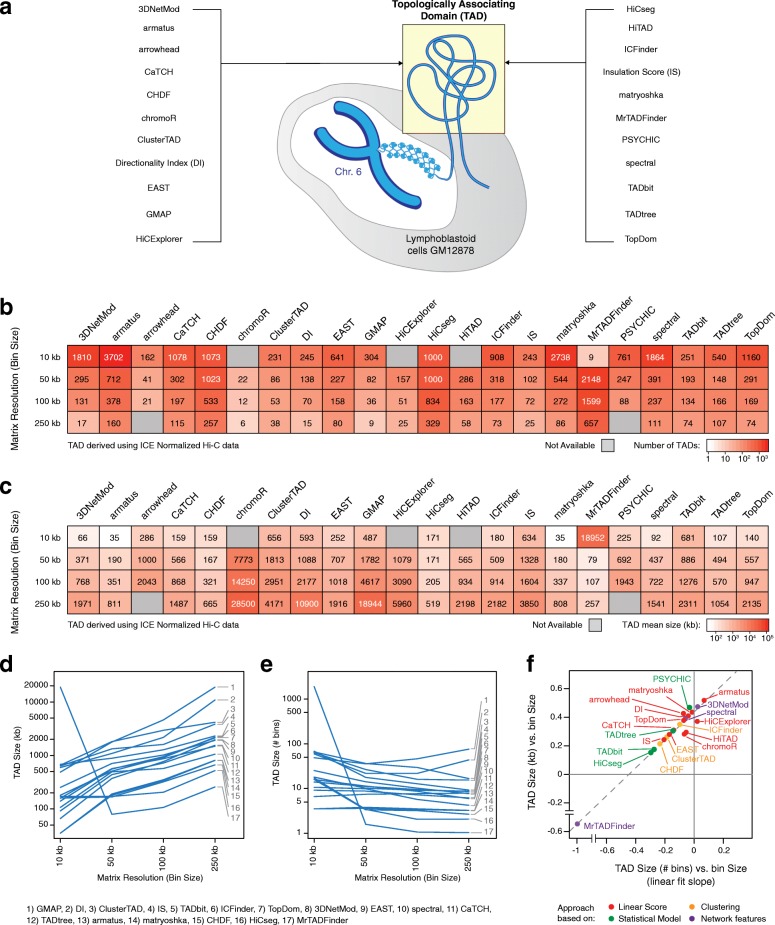
Identification of topologically associating domains (TADs) in chromosome 6 of the GM12878 cell line from ICE-normalized Hi-C data. **a** The performance of 22 TAD callers (listed on the left and right) was assessed using Hi-C data of the chromosome 6 of the lymphoblastoid cell line GM12878. **b** Total number of TADs detected in the ICE-normalized Hi-C data of chromosome 6 at four different resolutions (10, 50, 100, 250 kb) by each of the 22 TAD callers. Color intensity is proportional to the number of TADs in log-scale, and gray boxes correspond to TAD callers that did not successfully identified TADs at a given resolution. **c** Mean size (measured in kb) of the TADs detected in the ICE-normalized Hi-C data of chromosome 6 at four different resolutions (10, 50, 100, 250 kb) by each of the 22 TAD callers. Color intensity is proportional to the mean size of the TADs in log-scale, and gray boxes correspond to TAD callers that did not successfully identified TADs at a given resolution. **d**, **e** Variation of the mean size of the TADs measured in kb (**d**) or in number of bins (**e**) across Hi-C matrix resolutions. Each line refers to a TAD caller (numbered as indicated at the bottom of the plot), and only TAD callers that successfully identified TADs at all five resolutions are shown. **f** Slopes derived from the linear fit of the curves in panel **d** (TAD size in kb across resolutions) versus slopes derived from the linear fit of the curves in panel **e** (TAD size in number of bins across resolutions). Dots are colored based on the general approach used by the tool (“linear score,” “clustering,” “statistical model,” “network features”—see Table 1). The dashed line indicates the linear fit

In this study, we compared the performance of 22 TAD callers (Table 1 and Additional file 1), each on 20 different conditions (4 map resolutions each normalized with 2 independent strategies, plus 12 additional contact maps with variable sequencing depth), and assessed their results in terms of robustness to variable data resolution and normalization, concordance of the results among different callers, and ability to recapitulate biological features frequently associated with TADs and TAD boundaries. For callers identifying nested TADs, we separately investigate the enrichment of TAD-associated biological features at different levels of nesting. Each caller was run on the high-quality Hi-C data generated for chromosome 6 of the lymphoblastoid cell line GM12878 (Fig. 1a). This model was chosen as it is one of the cell lines where Hi-C data have been generated at the highest resolution [2], and chromosome 6 had the average chromosome length and number of reads per kb. Results were often heterogeneous among callers and conditions, prompting caution when drawing conclusions from specific TAD partitions, but also highlighted consistent good performance for some callers. Finally, results have been validated on 4 additional chromosomes of variable length and, for 6 selected callers, on additional datasets including independent replicates of the GM12878 cell line, human fetal fibroblast (IMR90), and mouse cortical neurons (herein referred to MCN). Overall, this study provides a useful reference to improve both future method development and the design of studies based on generation and analysis of Hi-C data.

**Table 1:** TAD callers analyzed in this study

TAD caller	Approach	# parameters	Hierarchical domains	Inter-TAD gaps
3DNetMod	Network features	18	Overlapping	Yes
armatus	Linear score	1	Overlapping	No
arrowhead	Linear score	1	Overlapping	Yes
CaTCH	Linear score	0	Nested	No
CHDF	Clustering	2	Disjoint	No
chromoR	Linear score	2	Disjoint	No
ClusterTAD	Clustering	2	Disjoint	No
Directionality Index (DI)	Linear score	3	Disjoint	Yes
EAST	Linear score	0	Disjoint	Yes
GMAP	Linear score	0	Nested	Yes
HiCExplorer	Linear score	5	Disjoint	Yes
HiCseg	Statistical model	3	Disjoint	No
HiTAD	Linear score	0	Disjoint	Yes
ICFinder	Clustering	2	Disjoint	No
Insulation Score (IS)	Linear score	5	Disjoint	Yes
matryoshka	Linear score	1	Nested	Yes
MrTADFinder	Network features	1	Disjoint	No
PSYCHIC	Statistical model	1	Nested	No
spectral	Network features	2	Disjoint	No
TADbit	Statistical model	0	Disjoint	No
TADtree	Statistical model	6	Overlapping	No
TopDom	Linear score	1	Disjoint	Yes

## Results

### TAD identification across data resolutions and normalizations

Data generated by high-throughput sequencing of chromatin conformation capture experiments (Hi-C) is typically organized in contact matrices, where interactions are grouped into bins of a predefined size and the numbers of contacts between pairs of bins constitute the entries of these matrices [1]. While the choice of the bin size is somewhat arbitrary and dependent of the sequencing depth, proposed heuristics select the minimum size such that the total number of reads per bin is deemed sufficient for a robust estimation of the contacts between each pair of loci [2]. Small bin sizes are possible only with high sequencing depth and allow for high-resolution analyses of chromatin loops. Conversely, large bin sizes might be used to study large chromatin structural elements (e.g., compartments [2, 5]) or are the necessary choice when a low number of reads are available leading to low-resolution matrices. As a consequence, the bin size of a Hi-C matrix reflects its resolution. Furthermore, values within such contact matrices are typically normalized to account for technical and experimental biases such as variable sequencing depth, GC content, fragment length, and sequence mappability. For the purpose of our analyses, we generated contact matrices for chromosome 6 of the GM12878 cell line with 4 different resolutions by subsampling reads from the original dataset such that the minimal bin size that guaranteed 1000 reads in at least 80% of the bins [2] was either 10 kb, 50 kb, 100 kb, or 250 kb. Then, we normalized each contact matrix using two popular approaches: the iterative correction and eigenvector decomposition [39] (ICE) and a parametric model of local genomic features [40] (LGF, a.k.a. HiCNorm). In total, we tested 22 callers, each across 8 different conditions (Fig. 1a).

First, we examined the number of TADs and their average size obtained with each TAD caller at each matrix resolution using ICE-normalized data. Not all callers successfully completed their run at all resolutions, with certain callers not performing well either with the smallest bin size (chromoR, HiCExplorer, HiTAD, and MrTADFinder) or with the largest bin size (arrowhead and PSYCHIC) (Fig. 1b, c—gray boxes). Interestingly, for a given bin size, the number of identified TADs could vary of up to two orders of magnitude among callers (Fig. 1b). Consequently, so did vary the average TAD size measured in number of base pairs (Fig. 1c). Similar results were obtained with LGF-normalized data (Additional file 2: Figure S1a). For both normalization procedures and examining TAD callers that returned results at all resolutions (*n* = 17), we found that the mean TAD size was generally increasing with increasing bin size (Fig. 1d and Additional file 2: Figure S1b). Conversely, the size of TADs measured in number of bins was relatively stable (Fig. 1e and Additional file 2: Figure S1c) for most of the callers, suggesting these callers tend to call TADs with the same number of bins, rather than corresponding to the same genomic region. In particular, the growth trends exhibited by TAD size as a function of bin size were remarkably similar independent of the normalization strategy adopted (Additional file 2: Figure S1d).

The slopes corresponding to the growth of the TAD size, measured by number of either base pairs or bins, based on the bin size adopted confirmed this tendency among all callers (Fig. 1f). Indeed, for almost all callers, we found positive slopes when the TAD size was measured in base pairs, whereas slopes were negative and close to zero when the TAD size was measured in number of bins. Exceptions were the Directionality Index (DI) and MrTADFinder, although TAD sizes estimated by the former did not vary monotonically and the latter performed poorly with bin size equal to 10 kb affecting the slope estimation. Interestingly, tools based on a linear score (Fig. 1f, red dots) had a greater tendency for identifying TADs with the same number of bins, rather than covering the same genomic regions, than tools relying on clustering-based approaches or statistical models (Fig. 1f, yellow and green dots, respectively).

Given different bin sizes corresponded to Hi-C contact matrices with a different number of reads, i.e., different resolutions, we wondered whether TADs observed in these different matrices reflected nested domains, such that the smallest domains are detectable with small bin sizes, whereas only large domains comprising the smallest ones can be detected with large bin sizes. If so, then TAD boundaries detected with large bin sizes must be a subset of the boundaries detected with small bin sizes (Additional file 2: Figure S2a). We tested this hypothesis for all TAD callers and computed the fraction of boundaries detected with a given bin size that contained at least one of the boundaries detected with the bin size immediately smaller (Additional file 2: Figure S2b). The results were overall variable among callers, but for several, we found that at least 50% of the boundaries were retained between resolutions. Notably, although arrowhead, GMAP, and PSYCHIC detect by default nested or overlapping TADs, their rate of boundary conservation was typically below 50% (Additional file 2: Figure S2b).

The presence of nested TADs can therefore explain, at least in part, the highly variable TAD size observed with different bin sizes. Nonetheless, boundary conservation was often low (below 50%) and size variability among callers for a given bin size remained pronounced. Overall, these findings challenge the possibility of defining a “typical TAD size” and indicate that the average size of a TAD is largely dependent of the tool used to identify these domains and resolution of the contact matrix.

To assess the similarity between TADs identified by the same caller using different normalizations or bin sizes, we recurred to the Measure of Concordance (MoC), previously introduced to compare clustering partitions [41]. Briefly, given two sets of TADs, MoC assesses the overlap between each pair of TADs, measured in number of base pairs and considering the overall size of both TADs (Additional file 2: Figure S2c). MoC ranges from 0, complete lack of concordance, to 1, perfect concordance, and it has the desirable property of being symmetric.

**Fig. 2:**
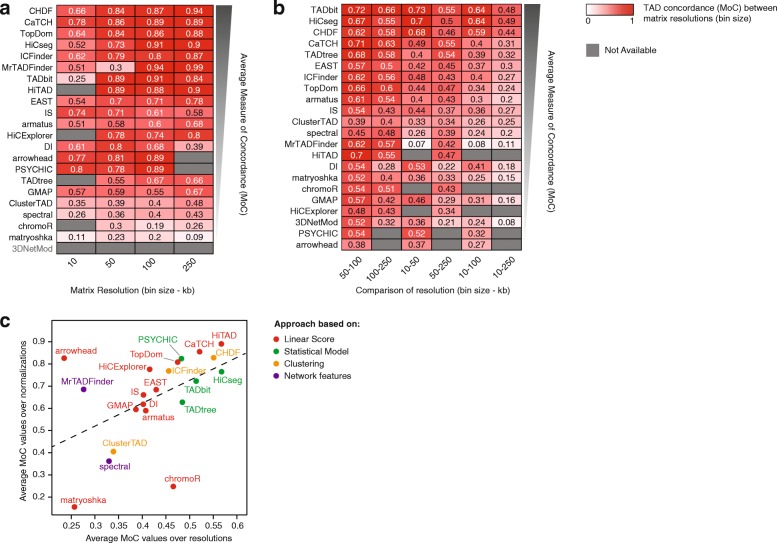
Concordance of TADs identified using different normalization strategies and resolutions. **a** Concordance between TAD partitions obtained with each TAD caller from ICE- and LGF-normalized matrices at five different resolutions (10, 50, 100, 250 kb) was assessed using the Measure of Concordance (MoC). MoC varies from 0 (absence of concordance, white) to 1 (full concordance, dark red). TAD callers are ranked based on the average of the MoC values across all resolutions (from highest to lowest). TAD callers that did not successfully identified TADs at a given resolution (gray boxes) were scored as 0 for the purpose of ranking by average MoC. **b** Concordance between TAD partitions obtained at different resolutions was assessed in a pairwise manner (e.g., 10 kb vs. 50 kb, 10 kb vs. 100 kb, etc.; results for the ICE data only are shown here) using the MoC. MoC varies from 0 (absence of concordance, white) to 1 (full concordance, dark red). TAD callers are ranked based on the average of the MoC values across all comparisons (from highest to lowest), and resolution comparisons are ordered according to the fold difference between matrix resolutions. TAD callers that did not successfully identified TADs at a given resolution (gray boxes) were scored as 0 for the purpose of ranking by average MoC. **c** Concordance across normalizations (average MoC obtained by comparing ICE and LGF partitions at different resolutions) versus concordance across resolutions (average MoC obtained by comparing Hi-C matrix of different resolutions for a fixed normalization. The dashed line indicates the linear fit

First, we compared TADs determined by each caller at a given bin size using ICE or LGF normalization strategy. Overall, TADs were often highly concordant between normalizations (MoC > 0.75) for about half of the callers (Fig. 2a), although results from ClusterTAD, spectral, chromoR, and matryoshka seemed particularly sensitive to the applied normalization strategy. We could not run 3DNetMod on LGF-normalized data, hence could not make the comparison for this caller. The concordance between TADs determined at different bin sizes was lower than it was between normalization strategies in all callers (Fig. 2b). In particular, as the ratio between the compared bin sizes increased, the concordance between the resulting sets of TAD decreased and almost invariably fell below 0.5 for five- to ten-fold bin size ratios (Fig. 2b). MoC values obtained by each caller between normalization strategies correlated with values obtained between different bin sizes (Fig. 2c). Overall, HiTAD, CHDF, CaTCH, TopDom, TADbit, PSYCHIC, and HiCseg demonstrated robust and consistent TAD partitions independent of the normalization and bin size adopted (Fig. 2c, top right corner).

Given many of these approaches rely on different parameters, we wondered how much the choice of these parameters influences the results. We should note that all callers have here been run using recommended parameters or default ones if no recommendations were made (see Additional file 1). For a subset of them, we compared the concordance between TADs identified using recommended or other parameters, but same bin size. Overall, most tools returned concordant TAD partitions (MoC > 0.7, Additional file 3: Table S1), with the exception of the Directionality Index (MoC > 0.6 for two independent comparisons), and Insulation Score (MoC = 0.32 and MoC = 0.4 for two independent comparisons). Both DI and IS have window size parameters that are highly dependent on the bin size adopted and, indeed, in our analyses we tuned these parameters when changing bin size (Additional file 1). These tests seem to indicate that parameter optimization is not a strict requirement for most tools. However, testing the robustness of a caller to its parameters might sometimes be necessary and callers with few or no parameters are preferable in this regard (see Table 1).

**Fig. 3:**
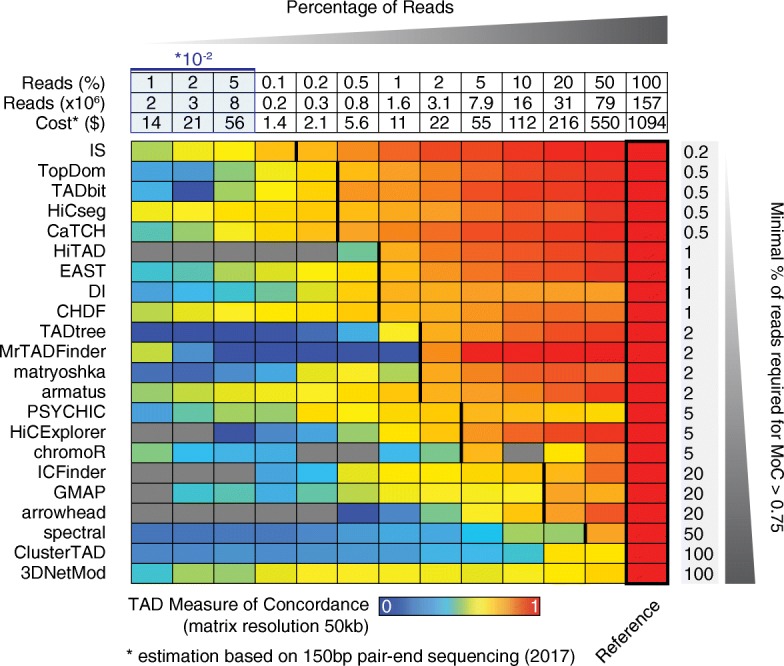
Concordance of TADs identified with different sequencing depths. Top panel (table). From the top: percentage of reads retained, actual number of reads (million), and estimated cost for generating the corresponding number of reads based on 150-bp paired-end sequencing in 2017. Bottom panel (heatmap). Measure of Concordance (MoC) values between TADs identified using 100% of the reads (rightmost column contoured in black) and TADs identified using a given percentage of reads (columns) for each TAD caller (rows). MoC values are color-coded with cold colors indicating low values (blue = 0) and warm colors indicating high values (red = 1). Percentages of reads used increase from left to right (see top panel for detailed values). Gray boxes correspond to TAD callers that did not successfully identify TADs with a given number of reads. Hi-C matrices were ICE-normalized and binned at 50 kb. TAD callers are ranked (from top to bottom) based on the minimal percentage of reads (values are reported on the right) required to identify TADs obtaining a MoC of at least 0.75 when compared to TADs identified using 100% of the reads

The ability of a caller to robustly perform independent of the resolution and quality of the data is highly desirable, as Hi-C experiments with high sequencing depth remain costly and, thus, difficult to scale to large datasets. To specifically assess the performance of all callers when different numbers of reads are available, independent of the bin size of the matrix, we systematically subsampled Hi-C contacts determined for the GM12878 cell line (chromosome 6) and compared the TADs called with subsampled reads with those obtained from the complete contact matrix. For this test, we used ICE-normalized data binned in 50-kb intervals and generated 13 different matrices using different percentages of reads that ranged from 100 to 0.01%, corresponding to an estimated cost ranging from over $1000 to less than a dollar (Fig. 3 top panel). Some of the tested callers failed to return a TAD partition for low sequencing depth, in particular arrowhead and HiTAD did not identify TADs with less than 1% of the reads (Additional file 2: Figure S3). Interestingly, the number of TADs and TAD size determined by each caller across subsampling (Additional file 2: Figure S3) were nonetheless less variable then they were when changing the bin size of the matrix (Fig. 1b, c). In agreement with our results across normalizations and bin sizes, TopDom, TADbit, HiCseg, and CaTCH all demonstrated highly concordant results: TADs generated with these callers using the full set of contacts were highly reproducible using less than 1% of the reads (MoC > 0.75) (Fig. 3). Notably, the best performance was here achieved by the Insulation Score (IS) that obtained MoC values > 0.75 with up to 0.2% of the total number of reads (Fig. 3) in agreement with recent analyses [42]. Using these callers would therefore lead to similar results at a fraction of the cost.

### Comparison of TADs identified by different callers

Up to this point, we have evaluated the concordance and robustness of the results obtained by each individual caller using different bin sizes and normalization strategies. Next, we compared the TADs obtained using different callers to assess their concordance. To this purpose, we fixed the bin size to 10 kb, corresponding to the matrices with the highest resolution, and used only ICE-normalized data, given the high concordance of results obtained with two normalization strategies for most callers. Using these settings, we could compare results from 18 callers.

**Fig. 4:**
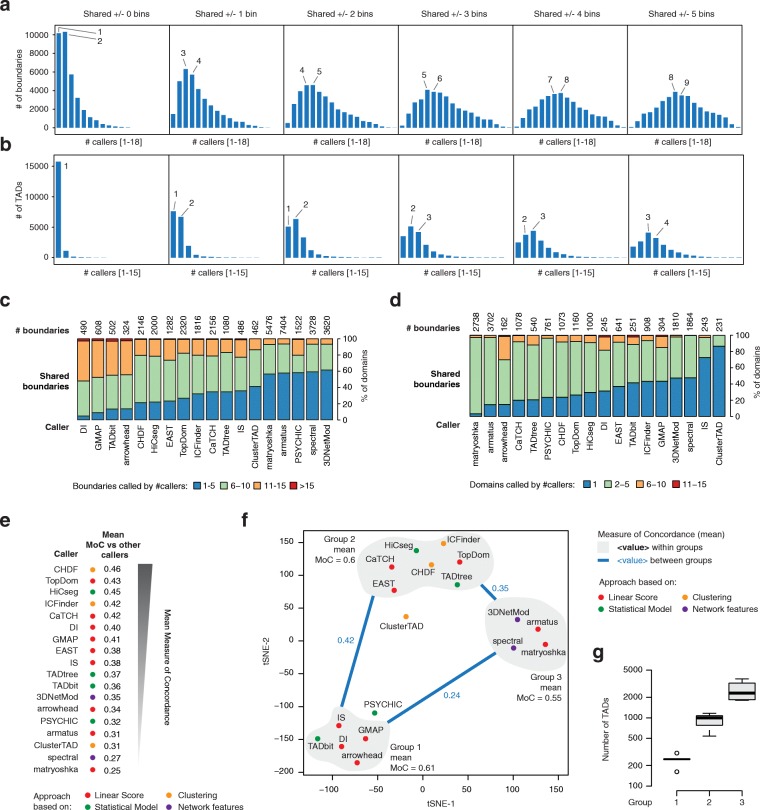
Pairwise comparison of TADs identified by all TAD callers (ICE-normalized Hi-C data at 10-kb resolution). **a** Histograms of the numbers of unique TAD boundaries (start and end positions of each TAD) identified by a given number of TAD callers (on the rows) with an increasing tolerance radius ranging from 0 (± 0 kb) to 5 bins (± 50 kb). **b** Histograms of the numbers of TADs identified by a given number of TAD callers (on the rows) with an increasing tolerance radius ranging from 0 (± 0 kb) to 5 bins (± 50 kb) for each TAD boundary. **c** Fraction of TAD boundaries identified by each TAD caller (rows) that were also identified by 5 or less (blue), between 6 and 10 (green), between 11 and 15 (orange), and more than 15 (red) other TAD callers. **d** Fraction of TADs identified by each TAD caller (rows) that were also identified by 1 (blue), between 2 and 5 (green), between 6 and 10 (orange), and more than 10 (red) other TAD callers. **e** Average Measure of Concordance (MoC) between TADs identified by each caller (rows) versus TADs identified by all other callers. TAD callers are annotated by the general approach they adopt (colored dots) and ranked (from top to bottom) by decreasing average MoC. **f** Map of the t-SNE analysis performed on the Pearson’s correlation matrix of the matrix of pairwise MoC between TADs identified by all callers. TAD callers are annotated by the general approach they adopt (colored dots). Three clusters were manually annotated, and the mean MoC within and between each of these groups is reported. ClusterTAD and PSYCHIC were not included in any of the clusters as their MoC values with each member/most members of closest group were below the average of the group. **g** Boxplot of the number of TADs detected by the callers in each of the three groups identified from the t-SNE analysis

First, we explored how frequently the same TAD or TAD boundary was called by different TAD callers (*shared* boundaries/TADs), using variable minimum distances between boundaries to determine shared TADs and TAD boundaries. Across all TAD callers, most of the boundaries were detected by less than half of the methods (Fig. 4a), and even less shared were TADs, with the highest fraction of them being detected by less than 4 callers (Fig. 4b). As expected, with increasing minimum distance, the fraction of shared TADs and TAD boundaries increased with distributions centered around 8 and 4 callers, respectively, for a minimum distance of 5 bins (± 50 kb). TADs and TAD boundaries detected by each caller exhibited different extent of agreement. Imposing a minimum distance of 2 bins (± 20 kb), most of the callers found at least 50% of boundaries that were also detected by more than 5 other callers (Fig. 4c) and this percentage was greater than 80% for the Directionality Index approach (DI), GMAP, TADbit, and arrowhead. Conversely, the majority of the boundaries called by matryoshka, armatus, PSYCHIC, spectral, and 3DNetMod were detected by less than 5 callers. It should be noted that, with the exception of PSYCHIC, these callers also identify a large number of boundaries (Fig. 4c). Consistently, DI, GMAP, arrowhead, and TADbit also identify the highest proportion of TADs that were also called by more than 5 other callers, whereas the majority of TADs called by IS and ClusterTAD were never detected by the other callers (Fig. 4d).

Next, we compared results from each pair of callers by Measure of Concordance (MoC). Average pairwise MoC values identify CHDF, TopDom, HiCseg, ICFinder, and CaTCH as the callers with highest mean MoC (MoC > 0.4) (Fig. 4e). Notably, these callers also scored among the most robust across bin sizes and normalization strategies (Fig. 2c–e). Stochastic neighbor embedding analysis (t-SNE [43]) of MoC values obtained by each caller against the others revealed three major groups of callers exhibiting high MoC values within group and low among groups (Fig. 4f). Callers within each group were often based on different algorithmic strategies; nonetheless, the number of TADs found by each caller was similar within group, but significantly different among them (Fig. 4g). Highly correlated pairwise MoC values were detected when comparing TAD partitions determined with bin size of 50 kb (Pearson’s correlation = 0.6, *p* value = 5E−33). To assess the concordance among TADs called by different methods in an independent manner, we computed the ratio between conserved TADs among each pair of callers and the minimum total number of TADs found by the two callers, i.e., the maximum possible number of TAD that can be conserved between the pair. The resulting mean conservation ratios were correlated with the mean MoC for all callers except for armatus, matryoshka, and arrowhead, which were the top three scoring based on this new analysis (Additional file 2: Figure S4a). Interestingly, the former two found by far the largest number of TADs; thus, even a small fraction of conserved TADs could still represent a relatively large number of domains, whereas arrowhead found the smallest number of TADs, indicating a high fraction of these domains were also called by other methods. Overall, these findings provide a reference to anticipate the concordance of the results based on specific TAD partitions depending on the adopted caller.

### TAD enrichment for CTCF and cohesin binding and histone methylation marks

While robustness and concordance are important features to assess the performance of a given algorithmic approach, the quality of its output is ultimately assessed by whether the results recapitulate true features of the system being analyzed. However, here, we lack a “true” set of TADs that could be used as a reference. Furthermore, the highly variable number of TADs and TAD sizes across callers challenge the design of synthetic datasets where a true TAD partition is pre-defined. To overcome these limitations, we evaluated the biological relevance of the TADs identified by each TAD caller, by assessing specific biological features that have been found frequently associated with TADs and/or TAD boundaries.

**Fig. 5:**
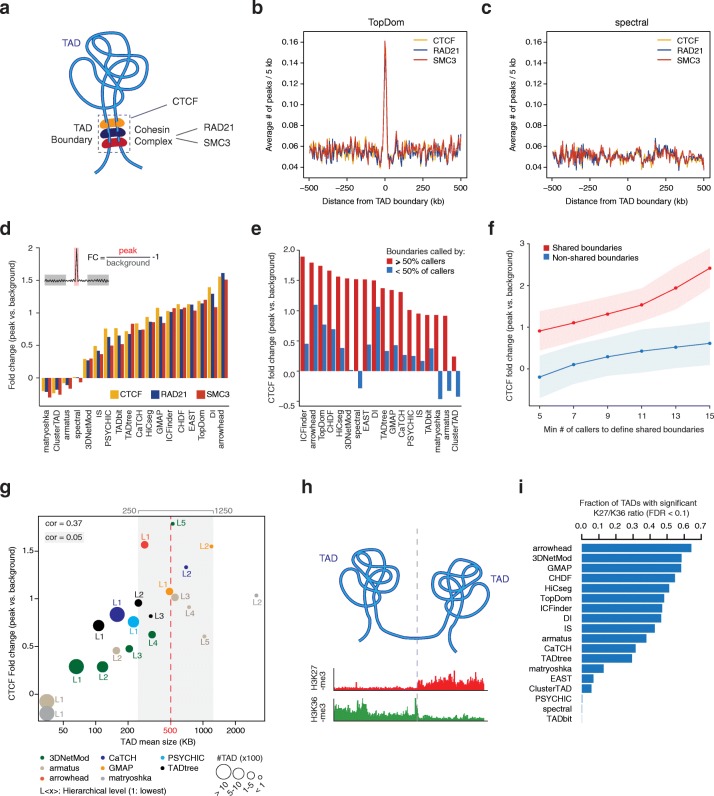
Assessment of TAD calling with biological features. **a** Schematic representation of the structural proteins CTCF (orange), RAD21 (blue), and SMC3 (red) that are enriched at TAD boundaries. **b**, **c** Representative examples of ChIP-seq peak signals (average number of peaks in 5-kb intervals) for TopDom (**b**) and spectral (**c**). Peak signals for CTCF (orange line), RAD21 (blue line), and SMC3 (red line) are overlaid. **d** Fold change of structural protein peak signals at TAD boundaries for CTCF (orange bar), RAD21 (blue bar), or SMC3 (red bar). TAD callers are ordered from left to right by increasing average fold change of peak signals of the three proteins. The fold change was computed as the ratio of protein binding signal at TAD boundaries (upper-left, red area) versus flanking regions (upper left, gray areas) minus 1. **e** Fold change of CTCF peak signal for boundaries called by at least 50% of the callers (red bars) or less than 50% of the callers (blue bars). **f** Mean fold change across callers of CTCF peak signal for shared (red track) and not shared (blue track) boundaries as a function of the minimum number of callers to define shared boundaries. Error areas correspond to one standard deviation. **g** CTCF fold change vs TAD mean size for each hierarchy level of the different callers. Hierarchical levels are labeled by increasing numbers (L1, …, Ln) with L1 being the level including TADs that do not contain nested TAD. Overall Pearson’s correlation = 0.37, Pearson’s correlation within the window [250–1250 kb] (gray area) is 0.05. The size of the dots is proportional to the number of TADs. **h** Schematic representation of H3K36me3 (green) or H3K27me3 (red) histone mark ChIP-seq read counts observed within TADs: TADs are typically enriched either for H3K36me3 marks (example of the left) or for H3K27me3 marks (example on the right) in a mutually exclusive manner. **i** For each TAD caller, fraction of TADs with a significant (high or low) H3K27me3/H3K36me3 log10-ratio (FDR < 0.1)

The chromatin insulator protein CTCF and the cohesin complex have been frequently reported at TAD boundaries, and they seem to be required for boundary formation [2, 6, 44] (Fig. 5a). Thus, we tested whether TAD boundaries identified by each caller were enriched for binding of these proteins. Peaks for CTCF, RAD21, and SMC3 were determined from chromatin immuno-precipitation followed by high-throughput sequencing (ChIP-seq) data and we explored the intensity of these peaks at domain boundaries and flanking regions. For some callers, e.g., TopDom, peaks of CTCF, RAD21, and SMC3 were pronounced at domain boundaries (Fig. 5b), whereas for others, e.g., spectral, peaks of the 3 proteins did not show any association with the TAD boundaries identified by the caller (Fig. 5c). To quantify these trends, we computed the percentage of TAD boundaries that were tagged by either of the 3 proteins (Additional file 2: Figure S4b) and the fold change between peaks found at TAD boundaries and those at adjacent flanking regions (Fig. 5d). Results from these analyses were correlated and consistent for all 3 proteins and identified arrowhead, DI, TopDom, and CHDF among the top 5 callers based on both criteria, with greater than twofold increase of CTCF binding signal at TAD boundaries and ~ 40% or more boundaries tagged by either CTCF or cohesin. Vice versa, matryoshka, ClusterTAD, armatus, and spectral always scored at the bottom with fewer than 20% of the boundaries tagged by either CTCF or cohesin and exhibiting no different ChIP-seq peak intensities between boundaries and flanking DNA regions (Additional file 2: Figure S4b and Fig. 5d). Given the variable level of agreement among TAD boundaries called by different methods, we tested CTCF enrichment at boundaries called by at least 50% of the callers (*n* = 9) compared to boundaries called by less than half of them. For all methods, we found a dramatic increase of CTCF fold changes for boundaries called by 50% or more callers (Fig. 5e) and this trend increases with the minimum number of callers consistently calling a boundary (Fig. 5f). Overall, shared boundaries always exhibited higher CTCF ChIP-seq signal than adjacent regions and the more callers identified a boundary, the higher the fold change of the signal (Fig. 5f).

Given some TAD callers can identify hierarchy of TADs or overlapping TADs (not necessarily nested—see Table 1), we investigated CTCF binding at TAD boundaries based on their nesting level. For this analysis, we only retained perfectly nested TADs and nesting levels comprising at least 10 TADs. Moreover, we define as the “lowest” nesting level (or L1), the set of TADs that do not contain any other nested TAD. In general, CTCF signal fold changes increased with the level of nesting, especially for tools that at the lowest level identified a very high number of small TADs (armatus, matryoshka, and 3DNetMod) and was positively correlated with the TAD mean size (Pearson’s correlation = 0.37). However, for mean sizes between 250 kb and 1.25 Mb, this correlation was no longer observed (Pearson’s correlation = 0.05) and results were thus independent of the TAD size (Fig. 5g—gray area). With increasing nesting, the number of TADs identified by each caller rapidly decreased. This might be a desirable feature for tools such as armatus and matryoshka that initially identified > 2500 TADs each. For these approaches, higher nesting levels led to a smaller number of TADs better associated with CTCF binding, even though CTCF fold changes remained consistently smaller than those observed for top scoring tools, such as arrowhead, DI, and TopDom. On the other hand, best CTCF fold change for GMAP and CaTCH (both obtained at L2—Fig. 5g) were derived from 87 TADs (mean size = 1 Mb) and 34 TADs (mean size = 550 kb), respectively, suggesting that these are not representative of the entire chromosome and lower nesting levels should also be considered to explore the full organization of the chromatin into TADs.

Histone-3 methylation marks are indicative of transcriptional activity within specific regions of the genome. Interestingly, it has been shown that TADs are frequently enriched for either activating (H3K36me3) or repressing (H3K27me3) marks [2, 7]. Hence, we explored the ratio between H3K27me3 and H3K36me3 within each TAD found by each caller and determined the percentage of TADs that exhibited a significant enrichment for either mark (empirical FDR < 0.1, see the “Methods” section) (Fig. 5h). Arrowhead was the top scoring approach also in this analysis, indicating that domains identified by this caller most frequently exhibit TAD-associated biological features (Fig. 5i). 3DNetMod, GMAP, CHDF, and HiCseg all identified more than 50% of TADs with a significant enrichment for either H3K36me3 or H3K27me3, whereas EAST, ClusterTAD, PSYCHIC, spectral, and TADbit scored at the bottom of this analysis, with less than 10% of the identified domains enriched for either of the histone marks (Fig. 5i). The association between TADs and H3K27me3 and H3K36me3 was largely independent of the nesting levels, for tools that identified nested TADs (Additional file 2: Figure S4c).

Overall, the results from these analyses based on known TAD-associated biological features were correlated, yet several tools scored well in one and poorly in the other (Additional file 2: Figure S4d). Arrowhead always scored at the top, but also GMAP, CHDF, TopDom, DI, HiCseg, and ICFinder exhibited a good enrichment for both CTCF/cohesin binding and histone mark specificity (Additional file 2: Figure S4d) and these results were confirmed even when accounting for nested TAD structures found by specific tools.

### Validation on independent chromosomes and datasets

All the analyses presented so far have been run on chromosome 6 of the GM12878 cell line. To test the reproducibility of our results on additional chromosomes, we repeated key analyses on chromosomes 1, 3, 13, and 15, chosen to include both large and short chromosomes with high sequencing depth. Specifically, we correlated the results obtained on chromosome 6 with those derived on the other chromosomes for the following analyses: mean TAD size, TAD concordance between normalizations (ICE vs. LGF), TAD concordance among different bin sizes, TAD concordance among different callers, enrichment of CTCF and cohesin peaks at TAD boundaries, and H3K27me3/H3K36me3 ratios within TADs. Across all analyses, we could confirm highly correlated results (Additional file 2: Figure S5), indicating that these are robust with respect to and independent of the chosen chromosome.

**Fig. 6:**
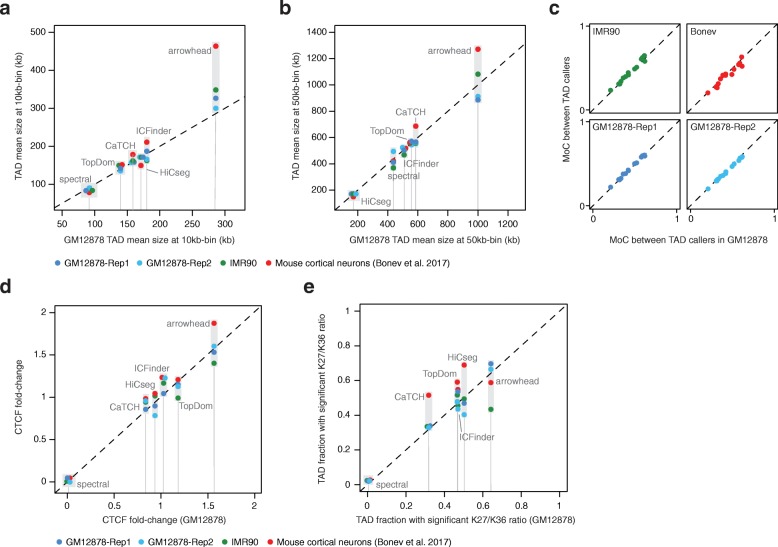
TAD caller performance across independent datasets. **a**, **b** Mean size of the TADs identified in the GM12878 Hi-C dataset (*X*-axis) versus the mean size of the TADs identified in four Hi-C datasets (*Y*-axis): GM12878 replicate 1 (dark blue), GM12878 replicate 2 (light blue), IMR90 (green), mouse cortical neurons (MNC, red). Results are shown for 6 TAD callers and Hi-C matrices using 10-kb bin size (**a**) and 50-kb bin size (**b**). For some of the TAD callers, jitter on the *X*-axis was added to improve visibility of the dots; the gray thin lines indicate the TAD mean size values in the GM12878 dataset. **c** Measure of Concordance (MoC) between TAD partitions determined by all pairs of callers (among the 6 tested—see panel **a**) in the GM12878 dataset (*X*-axis) and the other tested datasets (*Y*-axis). Results for each of these datasets are shown in distinct subplot; datasets are labeled and color-coded. **d**, **e** CTCF binding fold change (**d**) and ratios between H3K27me3 and H3K36me3 within TADs (**e**) obtained by the TAD callers for the GM12878 dataset (*X*-axis) versus and the corresponding values obtained in the four tested datasets (*Y*-axis, color-coded as in panels **a** and **b**). For some of the TAD caller, jitter on the *X*-axis was added to improve visibility of the dots; the gray thin lines indicate the TAD mean size values in the GM12878 dataset

To further corroborate our results, we compared TAD and TAD features derived from chromosome 6 of the GM12878 cell line with four distinct datasets: two independent replicates of the GM12878 model and two independent Hi-C datasets including human fetal fibroblasts (IMR90 cell line) and mouse cortical neurons (MCN) [45]. For these analyses, we selected a subset of 6 callers that exhibited variable performance across our tests: arrowhead, CaTCH, HiCseg, ICFinder, spectral, and TopDom. First, we compared the concordance (MoC) between TADs identified by each caller in the two independent GM12878 replicates. These datasets were separately generated, but derived from the same cell line; hence, a high level of concordance is expected. Indeed, all callers, except spectral, generated TADs with MoC greater than 0.7; for CaTCH, HiCseg, and TopDom, it was greater than 0.8. Next, we used the results derived from the complete GM12878 dataset as a reference and separately compared results from the GM12878 replicates, IMR90, and MCN against this reference. The mean TAD size identified by each caller was highly consistent between each pair of datasets, both using 10-kb and 50-kb bins, except for arrowhead that found bigger TADs in MCN compared to GM12878 (Fig. 6a, b). Nonetheless, the overall set of results were highly correlated, with spectral always identifying the largest number of TADs with smallest mean size, and arrowhead the smallest number with the largest size. Similarly, the concordance between TAD partitions determined by each pair of callers was remarkably consistent between GM12878 and the four other datasets (Fig. 6c). Finally, we compared CTCF binding fold changes and the ratio between H3K27me3 and H3K36me3 within TADs determined in the different experiments. Results for CTCF fold changes were highly correlated (Fig. 6d), whereas this correlation was weaker for the comparisons of histone mark ratios with IMR90 and MCN (Fig. 6e), potentially reflecting distinct specific histone mark changes associated with different cell types and species. Overall, results from independent replicates and datasets were highly concordant with those obtained for the GM12878, indicating that the performance of each method was largely independent of the analyzed dataset.

## Discussion

Topologically associating domains, or TADs, have recently been investigated across multiple biological contexts, to explore their role in both normal and disease development. The success and reliability of these studies depend however on the ability to correctly identify such domains. Here, we tested 22 distinct algorithmic approaches designed to detect TADs from Hi-C contact matrices. Our benchmarking study was designed to assess the results of each method on the same high-quality dataset based on its *robustness* to variable binning of the reads, data normalization, and sequencing depth; *concordance* of the results with those obtained by different methods; and *biological significance* of the identified chromatin domains.

We found that the number and, thus, mean size of TADs identified by different approaches on the same dataset are extremely variable. Furthermore, variation of the bin size used to generate the contact matrices led to different mean TAD sizes, even for the same approach. This variability suggests that it is difficult to propose a general definition of average TAD size, since this often depends on both the adopted method and data resolution. Interestingly, most callers identified large TADs when large bin sizes were used, and boundaries detected in these conditions were often a subset of those found with small bin sizes. These trends are consistent with a hierarchical architecture of nested TADs, rather than a single array of disjoint domains.

To summarize our results, we extracted top scoring methods based on them, satisfying five different criteria: robustness with respect to bin size and normalization strategy (Fig. 2); cost-effective performance, based on the ability of a caller to identify concordant TADs using less than 1% of the reads (Fig. 3); reproducibility of the results by other callers (mean MoC > 0.4) (Fig. 4); biological relevance of the results based on previously reported TAD-associated features (Fig. 5); and computational efficiency, defined by the maximal running time being below 10^3^ s (< 17 min) (Additional file 2: Figure S6). We found that TopDom, HiCseg, and CaTCH satisfied at least four out of five criteria (Table 2). It should be noted that TopDom and HiCseg do not identify TAD hierarchies or overlapping TADs. If sequencing costs are not a limiting factor, or to re-analyze available high-quality datasets, approaches like CHDF, ICFinder, and arrowhead would also be a suggested choice based on their performance on high-resolution matrices. In particular, at a 10-kb resolution, arrowhead was computationally efficient, scored at the top in both tests for enrichment of TAD-associated biological features, and it can output hierarchical TAD architectures. Importantly, caller performances and ranking were validated on distinct chromosomes, independent replicates of the GM12878 model, and independent datasets including different stages of cell differentiation (fetal lung fibroblast, IMR90) and different species (mouse cortical neurons).

**Table 2:** Result summary

Top scoring by:	Robustness to resolution and normalization	Cost-effectiveness	Concordance with other TAD callers	Enrichment for biological features	Computational efficiency
TopDom	*	*	*	*	*
HiCseg	*	*	*	*	
CaTCH	*	*	*		*
CHDF	*		*	*	
ICFinder	*		*	*	
TADbit	*	*			
arrowhead				*	*
GMAP				*	*
DI				*	
IS		*			
EAST					*

*TAD caller was among the top scoring in this test

## Conclusions

Over the past few years, the soaring generation of chromatin conformation capture experiments has led to the rapid development of several computational approaches to analyze and interpret this data. In particular, multiple methods have been developed to identify topologically associating domains (TADs) from the Hi-C contact maps. By systematically comparing the performance of 22 TAD callers, we highlighted poor concordance among the results generated by these callers at different resolutions, especially in terms of TAD numbers and sizes. Importantly, we found that this variability potentially reflects an underlying hierarchical domain organization that is only partially captured by different methods and at different resolutions. This observation indicates that approaches that allow the identification of hierarchical TAD structures might be preferable. However, we found that often TAD boundaries identified by tools that can detect such hierarchical structures were actually less conserved across experiments with different resolutions, than boundaries identified by tools that do not output domain hierarchies. Moreover, the use of some of these hierarchical TAD callers might be limited by high requirements of either data resolution (e.g., for arrowhead) or computational time (e.g., for TADtree) and, often, hierarchical levels return by these methods are few (2 or 3) with the number of TADs rapidly decreasing at each level. In conclusion, while strategies based on hierarchical TAD calling might ultimately be more appropriate to study chromatin domain architecture, current implementations need to be improved to reach broad applicability.

Overall, our work complements previous benchmarking studies [17, 18, 46] on Hi-C data analysis to provide a set of guidelines for the design of Hi-C computational and molecular studies and improve the robustness and reproducibility of their findings.

## Methods

### Hi-C data

Within this study, we used in situ Hi-C data for the GM12878 cell line generated by the Aiden-Lieberman group [2] (Gene Expression Omnibus accession number GSE63525). Lists of Hi-C contacts were downloaded, filtered for MAPQ ≥ 30 and merged across replicates. Intra-chromosomal lists of contacts were derived by extracting contacts between loci belonging to the same chromosome. The performance of the 22 selected TAD calling algorithms was extensively assessed using intra-chromosomal contact matrices generated for chromosome 6 of the GM12878 cell line, but key analyses were validated on chromosomes 1, 3, 13, and 15. Hi-C contact matrices were pre-processed with two different normalization strategies and at five different resolutions, for a total of 10 conditions for each caller. All custom scripts used in the analyses are available at https://github.com/CSOgroup/TAD-benchmarking-scripts.

#### Hi-C matrix normalization

Hi-C contact matrix normalization was performed using either the iterative correction and eigenvector decomposition method [39] (ICE) or a parametric model based on local genomic features (LGF) implemented by the HiCNorm method [40], using a Poisson regression model. ICE assumes equal visibility of each bin and considers that the observed interaction frequency can be written as a product of factorizable biases and “true” contact probabilities. ICE then proceeds through an iterative algorithm, dividing each row by its mean and updating the total vector of biases at each step. The procedure stops when the variance of the additional biases is negligible. In contrast, the LGF method explicitly models three systematic sources of biases (mappability, GC content, and restriction fragment length) using a Poisson regression that includes a parameter for each of these features. The normalized contacts are then the residuals of the fitted regression model.

Both approaches are implemented in the HiTC R package [47], respectively by the normICE (run with *max_iter=1000*) and normLGF (run with a Poisson regression model, *family=“poisson”*) functions.

Unlike ICE, LGF requires additional information to annotate each bin of the matrix with respect to the three bias features included in the regression model. Here, we used for this purpose the *getAnnotatedRestrictionSites* function from the HiTC package (with default parameters). For the restriction fragment length and GC content features, the function also needs reference genome as input (here derived from the *Bsgenome.Hsapiens.UCSC.hg19* R package). As for the mappability feature, a mappability file was retrieved from http://hgdownload.cse.ucsc.edu/goldenpath/hg19/encodeDCC/wgEncodeMapability/wgEncodeCrgMapabilityAlign100mer.bigWig.

#### Hi-C matrix resolutions

To generate intra-chromosomal Hi-C contact maps at various resolutions, we adopted a previously introduced definition of map resolution [2], i.e., the resolution of a contact map is given by the smallest bin size such that at least 80% of the bins make at least 1000 contacts. Based on this definition, we subsampled the intra-chromosomal list of contacts list such that the smallest bin size satisfying the above criteria was, respectively, 10 kb, 50 kb, 100 kb, 250 kb, and 1 Mb.

For the comparison of TAD partitions described in Fig. 3, we fixed the bin size at 50 kb and subsampled the complete intra-chromosomal list of contacts for decreasing percentages of reads. For each caller, TAD partitions determined from the subsampled Hi-C contact maps were compared to the partition obtained from the complete contact map by Measure of Concordance (MoC) (see the “Assessing concordance between TAD partitions” section).

### TAD callers

In total, we used and compared 22 TAD callers (see Additional file 1 for a description of each method and chosen parameters). Briefly, we categorize each caller in four groups based on the general approach adopted. The majority of the methods computes a *linear score* associated to each bin summarizing the distribution of contacts made by that bin. This group includes armatus, arrowhead, CaTCH, chromoR, DI, EAST, HiCExplorer, HiTAD, IS, matryoshka, and TopDom. A second group relies on *statistical models* of the interaction distributions and includes GMAP, HiCseg, PSYCHIC, TADbit, and TADtree. A third group uses instead *clustering* approaches applied to the Hi-C matrix and includes CHDF, ClusterTAD, and ICFinder. Finally, a fourth group of callers applies *network metrics* based on the concept of network modularity or connectivity to identify communities of interacting genomic loci; these are 3DNetMod, MrTADFinder, and spectral. As often as possible, we applied these tools with their default user-defined parameters or set these parameters to the values recommended by the authors in the corresponding manuscript or software support file (see Additional file 1 for details). The outputs of the different callers were converted to a uniform three-column format of 1-based genomic coordinates indicating for each TAD its chromosome, start position (i.e., starting genomic coordinate of the bin identified as left boundary), and end position (i.e., ending genomic coordinate of the bin identified as right boundary), e.g., chr1, 1, 10000; chr1, 10001, 20000; etc.

### Robustness to parameter tuning

Analyses with other parameter settings were run on 50-kb ICE-normalized Hi-C matrix (GM12878 dataset), except for DI and IS where the 10-kb matrix was used.

#### Arrowhead

Two additional settings for the size of the sliding window were tested: 1000 and 3000 [default 2000].

#### DI and IS

For the supplementary analyses, we ran DI and IS using two additional window sizes, 2.5 Mb and 5 Mb, that correspond to the window size setting described in the main text for the 50-kb and 100-kb bin sizes, respectively [default at 10-kb bin size is 500 kb].

#### HiCseg

In the main text, we used HiCseg with the model (“D”) that assumes that the means are constant outside the diagonal. For the supplementary analysis, we ran HiCseg under the assumption that the means are constant in bands outside the diagonal blocks (“Dplus” model).

#### ICFinder

For this caller, we tried varying the thresholds used for merging the clusters [default: *σ*− = 0.3, *σ*+ = 3,], changing them such that they define a broader or a narrower range: *σ*− = 0.1, *σ*+ = 6 and *σ*− = 0.6, *σ*+ = 2, respectively.

#### Spectral

Here, we tested different algebraic connectivity threshold: *λ* = 0.4 and *λ* = 0.9; default setting was *λ* = 0.8. This value is used as a stopping criterion in the recursion step of the spectral algorithm (upper bound of the Fiedler number).

#### TopDom

Two additional window sizes (“w”) were tested: 10 and 20; default was w = 5; recommended range is 5–20. This parameter defines the size of the window used to compute the binSignal.

### Assessment of TAD caller performance

#### Assessing concordance between TAD partitions

To compare TAD partitions, we adopted the Measure of Concordance (MoC), a metric introduced to compare clustering assignments [41]. The MoC is defined as follows:

$$ \mathrm{MoC}\left(P,Q\right)=\Big\{{\displaystyle \begin{array}{c}1 if{N}_P={N}_Q=1\\ {}\frac{1}{\left(\sqrt{N_P{N}_Q}-1\right)}\left({\sum}_{i=1}^{N_P}{\sum}_{j=1}^{N_Q}\frac{\parallel {F}_{i,j}{\parallel}^2}{\parallel {P}_i\parallel \parallel {Q}_{\mathrm{j}}\parallel }-1\right)\mathrm{otherwise}\end{array}}\operatorname{} $$

where *P* and *Q* are the sets of TADs being compared including *N*_*P*_ and *N*_*Q*_ TADs, respectively. *P*_*i*_ and *Q*_*j*_ are two individual TADs within *P* and *Q* having size ||*P*_*i*_|| and ||*Q*_*j*_||, respectively, measured in base pairs. Finally, ||*F*_*ij*_|| corresponds to the size (number of base pairs) of the overlap between the two TADs *P*_*i*_ and *Q*_*j*_.

The MoC is symmetric and upper and lower bounded, ranging from 0 (absence of concordance) to 1 (full concordance). The MoC was initially designed for the comparison of clustering assignments generated by distinct algorithms on the same number of elements. In the context of TAD calling, base pairs correspond to the elements being clustered, and the TADs to the clusters. Some, but not all, of the callers that we compared identify both TADs and inter-TAD regions, i.e., genomic regions that are in between TADs, but not called as TADs. To allow for a systematic comparison among all callers, we considered both TADs and inter-TAD regions as clusters when computing the MoC.

In our study, the MoC was used in four different contexts: for each caller separately, to quantify the concordance between partitions from ICE and LGF for each of the five resolutions (robustness to normalization, Fig. 2 a), to quantify the concordance between TADs called at different bin sizes (robustness to resolution, Fig. 2b), and to quantify the concordance between TADs called from subsampled Hi-C data (robustness to sequencing depth, Fig. 3). Finally, TAD partitions identified by different TAD callers were compared by MoC in a pairwise manner using ICE-normalized data binned at 10-kb resolution (Fig. 4e, f).

#### TAD and boundary conservation

Besides the Measure of Concordance, we also assessed the fractions of boundaries and TADs that were conserved among resolutions (Additional file 2: Figure S2) and callers (Fig. 4a–d). When TADs and TAD boundaries were compared between different callers, results were derived from Hi-C matrices at the same resolution, i.e., identical bin size. Here, a boundary *b*_1_ from caller 1 was called conserved if a boundary *b*_2_ was found by caller 2 within a radius of *x* bins from *b*_1_ with *x* being an integer value ranging from 0 to 5. A TAD found by caller 1 was called conserved if both its boundaries were conserved and they corresponded to the start and end positions of exactly one TAD found by caller 2.

To determine TAD boundary conservation between analyses run on Hi-C matrices at different resolutions, i.e., different bin sizes, we called conserved a boundary *b*_1_ found with the bigger bin size (e.g., 50 kb), if a boundary *b*_2_ was found with the smaller bin size (e.g., 10 kb) within the interval delimited by *b*_1_. As before, a TAD found by caller 1 was called conserved if both its boundaries were conserved and they corresponded to the start and end positions of exactly one TAD found by caller 2.

#### Comparison with ChIP-seq data for structural proteins

ChIP-seq peaks derived on GM12878 cells for CTCF and members of the cohesin complex, SMC3 and RAD21, were downloaded from ENCODE (www.encodeproject.org). For SMC3, we used peaks from experiment ENCSR000DZP. For RAD21, we used the intersection of peaks from experiments ENCSR000EAC and ENCSR000BMY. For CTCF, we used the intersection of peaks from experiments ENCSR000DKV, ENCSR000AKB, ENCSR000DZN, and ENCSR000DRZ. To determine the percentage of TAD boundaries tagged by a structural protein, we selected TAD boundaries having at least one ChIP-seq peak of the protein of interest overlapping with the boundary or at least one of the two adjacent bins (i.e., ± 10 kb from the boundary). To assess the differential binding of these structural proteins at TAD boundaries compared to non-boundary regions (Fig. 5d), we first determined a structural protein profile (“SPP”) at each boundary, by computing the average number of peaks in 5-kb intervals within the region surrounding the boundary (± 500 kb). Next, we computed the fold change between the average SPP in a narrow interval surrounding a TAD boundary (a.k.a. “peak”, ± 10 kb or 1 bin radius from the boundary) and the average SPP in two regions spanning 100 kb each and located 400 kb apart from the TAD boundary. For the analysis of shared boundaries, a boundary was called “shared” or “not shared” based on the number of callers *N* that identified that boundary (with a tolerance of two bins, as described before). Fold change of ChIP-seq peaks was computed for each category and for different *N* values.

#### Hierarchy level analysis

For each TAD caller giving nested or overlapping domains as output, we extracted each level of the hierarchy at 10 kb in the following way:

1. Level 1 was the TAD partition at 10 kb used in all the other analyses, such that no further nesting was found for TADs and level 1
2. Each TAD at every additional level *i* (*i* = 2,...,*N*) had to contain at least one domain (i.e., its start and end) present at level *i* − 1. TADs of level *i* that were coincident with TADs present at previous levels were discarded
3. Levels containing less than 10 domains were discarded from downstream analyses.

Specifically:

- For 3DNetMod, arrowhead, and TADtree, which were giving overlapping domains as output, we extracted levels 2 to *N* levels
- For armatus, we pooled the domains of suboptimal solutions and we extracted levels 2 to *N*
- For CaTCH, we used three additional RI cutoffs (0.7, 0.75, and 0.8) to derive domains for levels 2, 3, and 4 respectively, discarding invalid domains as explained above in points 2 and 3
- For GMAP, matryoshka, and PSYCHIC, we extracted the additional levels directly from the output, discarding invalid domains as explained above in points 2 and 3

#### Comparison with ChIP-seq data for histone marks

ChIP-seq signals (fold change over control, pooled replicates) for H3K27me3 and H3K36me3 derived on GM12878 were downloaded from ENCODE (experiments ENCSR000DRX and ENCSR000DRW, respectively). For each caller, we computed an interval size equal to 10% of the average size of the TADs (measured in base pairs) and grouped the ChIP-seq signals into equally spaced intervals of the calculated size. For each interval, we computed the log10-ratio between the H3K27me3 and H3K36me3 signals (LR values or LR intervals). Next, we computed the observed average LR values within each TAD and in order to evaluate the significance of these values, we shuffled 10 times the LR intervals and derived a distribution of randomized average within-TAD LR values. An empirical *p* value was derived for each TAD by comparing its observed average LR with the distribution resulting from the shuffling. Finally, empirical *p* values were corrected for false discovery rate (FDR) using the Benjamini-Hochberg procedure and for each caller we reported the fraction of TADs having an FDR corrected *p* value smaller than 0.1.

#### Computational performance

We assessed the computational performance of the TAD callers with two different metrics: the user time (running time) and the maximum resident set size (memory usage). Normalization and pre-processing steps were not taken into account. Timing and memory usage monitoring were retrieved with the Linux command *time*. All analyses were run on a x86_64-redhat-linux-gnu/Intel(R) Xeon(R) CPU E5-2699 v4 @ 2.20GHz.

### Validation on other chromosomes

Validation analyses were performed on chromosomes 1, 3, 13, and 15. Hi-C matrices for these chromosomes were processed as for chromosome 6, and TADs were detected using the 22 TAD callers (in case a TAD caller failed for one of the chromosomes, it was discarded from the comparison). Then, we relied on eight features to compare the results obtained on chromosome 6 with those derived for the other chromosomes in a pairwise manner:

1) *Mean TAD size*. For each TAD caller, we retrieved the average TAD size (in base pairs) of the TADs it identified and computed the Pearson’s correlation coefficients (PCC) between values obtained for chromosome 6 and those for each of the other tested chromosomes.

2) *TAD MoC across normalizations*. For each TAD caller, we computed the Measure of Concordance (MoC) between the partitions obtained for ICE and LGF, at a fixed resolution (e.g., TopDom 10 kb ICE vs. TopDom 10 kb LGF). Corresponding values obtained for each of these comparisons for chromosome 6 and for each of the other tested chromosomes were correlated using PCC.

3) *TAD MoC across resolutions*. For each TAD caller, we computed the Measure of Concordance (MoC) between all possible combinations of resolutions (bin sizes), for a fixed normalization (e.g., TopDom 10 kb ICE vs. TopDom 50 kb ICE). Corresponding values obtained for each of these comparisons for chromosome 6 and for each of the other tested chromosomes were correlated using PCC.

4) *TAD MoC across callers*. We computed the MoC between pairs of TAD callers for the Hi-C ICE-normalized data at 10-kb resolution. MoC values obtained for chromosome 6 were compared to those obtained for each of the other tested chromosomes.

5–7) *CTCF, RAD21, and SMC fold change*. For each TAD caller, we computed the fold change between the enrichment of CTCF (or RAD21 or SMC3) ChIP-seq peaks at TAD boundaries versus adjacent flanking regions. Fold changes obtained across the callers for chromosome 6 were then correlated with the ones obtained for each of the other tested chromosomes by PCC.

8) *Fraction of TADs with significant H3K27me3/H3K36me3 ratios.* For each TAD caller, we determined the fraction of TADs showing a significant (FDR < 0.1) log10-ratio between H3K27me3 and H3K36me3 signals, assessed with the procedure described in the section “Comparison with ChIP-seq data for histone marks.” Subsequently, we computed the PCC between values obtained for chromosome 6 and the ones obtained for each of the other tested chromosomes.

### Validation on other datasets

Hi-C data for replicates of GM12878 and IMR90 were downloaded from GSE63525, and CTCF ChIP-seq data were downloaded from ENCODE. Hi-C and ChIP-seq data for mouse cortical neurons were downloaded from GSE96107. All data were processed as described before, and key analyses outlined in the section “Validation on other chromosomes” were performed between chromosome 6 of the full GM12878 dataset and of the other datasets.

### Statistical analyses

All plots and statistical analyses were conducted on R version 3.3.1 (R Core Team 2016). t-SNE analysis was performed on the Pearson’s correlation matrix of the matrix of pairwise MoC between callers using the *Rtsne* function from the *Rtsne* R package [43] (with the parameters *dims = 2*, *perplexity = 5*, and *pca = FALSE*).

## Additional files

Additional file 1: Supplementary note providing a description of each TAD caller and parameters used in this study. (PDF 135 kb)

Additional file 2: Supplementary figures and figures legends. (PDF 359 kb)

Additional file 3: Supplementary Table S1. (XLSX 11 kb)
