# Supplementary material for: Comparison of computational methods for the identification of topologically associating domains
Source: Genome Biol. 2018 Dec 10;19:217. doi: 10.1186/s13059-018-1596-9 (PMC6288901; doi:10.1186/s13059-018-1596-9)
Supplement: Supplementary file 1 — Supplementary note providing a description of each TAD caller and parameters used in this study. (PDF 135 kb) [file 13059_2018_1596_MOESM1_ESM.pdf]

SUPPLEMENTARY NOTE 1

TAD calling methods (sorted by publication year) and parameters used in this study

Directionality Index[1] (DI) This approach defines a score, or directionality index, that quantifies the bias of each bin for interacting preferentially with upstream or downstream regions. An Hidden Markov Model is then used to predict three hidden states (upstream bias, downstream bias, no bias) and start and end of each TAD are inferred accordingly.

For this method, the main parameter is the size of the window used to calculate the DI. We set this parameter proportionally to the one used by the authors (2Mb for 40kb data resolution). Other internal parameters for the TAD calling (thresholds of median probabilities and minimal size for probability correction) were let to their default values (0.99 and 2, respectively).

Armatus (v2.2) [2] identifies non-overlapping domains that are persistent across multiple values of the gamma resolution parameter of the method (low gamma values lead to large domains, high gamma values lead to small domains). Precisely, for each gamma, a set of TADs are identified using an objective function based on the scaled density of the graph defined by a given domain. The consensus set of TADs that are persistent across resolutions and non-overlapping is then retrieved using a dynamic programming approach.

The following parameters are required: maximal resolution, step size for the resolution value and the minimal number of submatrices available to compute the mean in the quality function. We retained the default value of each parameter: 0.5, 0.05, and 100, respectively. In addition, as armatus can enumerate multiple optimal and near-optimal solutions, we restrict it to provide one single solution.

Arrowhead (Juicer v1.5.2) [3] applies an ad hoc transformation of the Hi-C matrix (arrowhead matrix) aimed at emphasizing TAD borders. TADs assume arrowhead features in this matrix and a corner score is defined as the sum of 3 scores: i) sum of the signs in lower triangle minus sum of the signs in upper triangle; ii) sum of the values in lower triangle minus sum of the values in upper triangle; iii) total variance in upper and lower triangle. Domain corners (i.e. boundaries) will be identified by detecting points with maximal score.

We set the window size parameter to its default value (2000).

chromoR (v1.0) [4] This method uses a wavelet change point analysis. Hi-C data are summarized into a 1D contact profile by taking the row sums of the contact matrix. Position of the change points (i.e. TAD boundaries) are detected using a wavelet Poisson change point detection algorithm. The final set of change points is obtained by taking those that maximize the fit with the data, evaluated by the Akaike's Information Criterion.

chromoR needs minimal (minL) and maximal (maxL) levels at which change points are searched. To perform the widest multi-scaled search, we set minL to its smallest possible value (1) and maxL to its largest possible value (which is imposed to not exceed 10% of the length of the profile).

HiCseg (v1.1) [5] Here, the authors define a statistical model for the Hi-C matrix (a block-wise segmentation model) for identifying TADs by retrieving boundaries of the diagonal blocks based on a maximum likelihood apprach, as in a typical 2D segmentation problem. This problem is then solved by dynamic programming (reduced to a 1D segmentation problem).

The maximal number of change points was set to 1000, the distribution of the data was set to Gaussian (as recommended by the authors for normalized data), and we used a block-diagonal model.

CHDF[6] considers that three types of regions constitute a Hi-C matrix: domain regions, regions between two adjacent domains (interaction between two domains), and residual regions. The first two are considered as the clusters and clustering is performed to detect the borders of the domains. As a clustering function, a sum-of-squared-error of interaction counts is computed for each kind of region over all the domains. The optimal solution is found using dynamic programming with an objective function combining this sum-of-squared-error criterion and a penalty term that allows to favor domain regions with higher numbers of interactions than intra-domain regions.

We set the maximal number of domains and their maximal size to 1000 and to the chromosome size, respectively.

Insulation Score (v1.0.0) [7] **(IS)** The algorithm defines for each bin in the diagonal of the Hi-C map an "insulation square", i.e. a squared region with the given bin as its lower vertex. The mean number of interactions in such square is defined as the insulation score (IS) of the corresponding bin. The IS vector is then converted into a delta vector that approximates the slope in the original IS vector. TAD boundaries are identified as the minima of the delta vector that further pass boundary strength filter.

Parameters were set as described by the authors: 500 kb for the insulation square and 250 kb for the insulation delta span at 10 kb data resolution (adjusted proportionally for other resolutions), 0.1 for the noise threshold, 3 for the boundary margin of error and "mean" as aggregation function.

Matryoshka[8] This approach is a further development of the armatus method (Filippova et al. 2014). A hierarchy of non-overlapping domains is built by detecting domains at different resolutions using armatus at each resolution. Non-overlapping domains at each resolution are clustered using the variation of information as distance metric, with the constraint of clustering domain sets at contiguous resolutions. A set of consensus domains is then derived at each level of the hierarchy.

Parameters were defined as for armatus.

Spectral[9] The authors here use an approach based on spectral graph theory to segment the chromosome from the Fiedler vector of the Laplacian of the Hi-C matrix. Upon computing the normalized Laplacian of the Hi-C map, the Fiedler vector is used to generate a first partition of domains, which are then recursively split until the Fiedler number of the obtained domain is higher than a chosen threshold or its size reaches a lower bound.

We set these the minimum Fiedler number to 0.8 (as used in the source code and in the corresponding article) and the minimal TAD size to 1 bin.

TADtree[10] identifies nested hierarchies of TADs from Hi-C maps. The TAD forest is detected by solving an optimization problem combining two criteria: 1) a linear model describing the enrichment of contacts within TADs, 2) the boundary index, i.e. a measure of the shift in interactions between bins that are upstream and downstream of a given bin.

Users need to define six parameters, that we chose as follows (as in the example control file, otherwise stated): p = 3, q = 12 (boundary index parameters), gamma = 500 (balance between boundary index and squared error in the score function), maximal size of a TAD (in bins): 50, maximal number of TADs in each tree: 25, maximal number of TADs: 1000, i.e. ~6 TADs/Mb as suggested by the authors and as used in (Forcato et al. 2017).

TopDom (v0.0.2) [11] computes for each bin a statistic (*binSignal*) based on the contact frequency among upstream and downstream chromatin regions. Boundaries of TADs are then identified as

minima of *binSignal* values along the chromosome using a piecewise linear function. False positives are then filtered by testing the difference between interactions within upstream and downstream regions and between such regions by Wilcoxon rank sum test.

To calculate the *binSignal*, the authors recommend using a window size between 5 and 20. We set this value to 5 as it was the one found the most appropriate in their article.

CaTCH (v1.0) [12] is an algorithm that uses a metric called reciprocal insulation to quantify how well a TAD is isolated from its neighbors. The method starts by segmenting the Hi-C map into a set of domains, and then merging consecutive TADs whose reciprocal insulation score is lower than a given threshold. In order to stratify the whole hierarchy of TADs, CaTCH systematically varies this latter value.

For the purpose of TAD caller benchmarking, we set this threshold to 0.65 as the authors report this was the value leading to maximal CTCF enrichment at boundaries. This value was also close to the value leading to the best match with DI (0.69).

ClusterTAD[13] uses an unsupervised clustering approach for the detection of TADs. Each bin [i,j] is clustered based on the interaction profiles of the corresponding i-th row and j-th column and using the K-means clustering method. The quality of the domains retrieved for different Ks is assessed by computing the difference of contact frequencies within TADs and among adjacent TADs. For a given chromosome, the quality score is the average of this value for the set of TADs and the partition with highest quality score is retained for the chromosome.

For ClusterTAD, the user should provide minimal and maximal TAD size limits (default values for 40kb Hi-C data: 120kb and 600kb respectively). As the default maximal size was not compatible with our larger bin size, the maximal size was rescaled proportionally to the bin size (e.g. 600kb for 40 kb bin size, 750kb for 50kb bin).

EAST (2.0) [14] is an algorithm that relies on 2D convolution of Haar-like features to retrieve TADs based on a scoring function quantifying the quality of a given genomic region. The summed area table (SAT) data structure is used to efficiently compute a weighted sum of pixel values for a particular location. This weighted sum is used to assess the quality of the domains by combining three terms that reflect the following properties: 1) high frequency of interactions inside the region; 2) low frequency with neighborhood; 3) higher frequency between the start and the end than the average inside. Dynamic programming is then used to find the optimal set of contiguous and non-overlapping domains that maximizes the sum of these domain scores.

Default parameter values are provided in the source code of EAST, no parameter needs to be chosen by the user.

GMAP (v.1.1) [15] proceeds in three steps: 1) a two-component Gaussian mixture model is used to describe chromatin interactions within and outside of a domain, 2) boundary bins are identified testing the fraction of observed interaction counts that are within regions flanking the bin and between such regions, 3) finally, TADs are called based on the boundaries detected in step 2. To detect a hierarchy of TADs, the same three steps are applied to each TAD to detect its subTADs.

Except for the resolution parameter (bin size of the input matrix), the GMAP R function was launched using the default parameters (logt = TRUE, dom_order = 2, min_d = 25, Max_d = 100, min_dp = 5, Max_dp = 10, Bg_d = 200, hthr = 0.9, bthr = Bg_d, t1thr = 0.75, fcthr = 0.9).

HiTAD (TADLib 0.3.1-r1) [16] starts by computing an adaptive directionality index (DI) that adjusts the window size to the local interaction environment and serves as input to an HMM to identify a set a boundaries. This step is repeated iteratively with new window sizes until convergence, generating a pool of "bottom domains". The best subset of boundaries are found by optimizing an objective function defined by the enrichment of intra-domains interaction frequencies versus inter-domain interaction frequencies. To identify a hierarchy of TADs, the optimization step is repeated using as input the subset of initial boundaries located within the domain.

A maximal TAD size has to be indicated for HiTAD. Here we used the default value (4000000 bp).

ICFinder[17] uses a hierarchical clustering approach for calling TADs. Starting by considering each column of a Hi-C matrix as a cluster, the algorithm iteratively merges closest clusters while imposing linear connectivity. The stopping rule for merging clusters relies on the heterogeneity (variance) of the candidate merged cluster (merging requires weak heterogeneity). In case of intermediate heterogeneity, an additional criterion based on a local directionality index is used to decide if a boundary has to be fixed.

ICFinder requires two parameters to be defined: the low (σ^-) and high (σ^+) variance thresholds. We used default values ($\sigma^- = 0.3$ and $\sigma^+ = 3$).

MrTADFinder[18] TAD detection is here formulated as a community detection problem in a graph, as TADs correspond to densely interacting networks of genomic loci. The authors define a null model as well as a modularity function adapted to Hi-C maps. The TADs are then identified by optimizing the modularity function using a modified Louvain algorithm, that takes into account that domains are made of continuous segments.

The objective function contains a resolution parameter that has to be set. No value was recommended by the authors, and we chose a value of 2.9 as this led to best match with the DI method.

PSYCHIC[19] was developed to identify promoter-enhancer interactions from Hi-C data. The algorithm employs a two-component probabilistic model to estimate the probability of intra- and inter-TAD interactions. Free parameters of the model are estimated from segmentation of Hi-C maps obtained using the Directionality Index (DI). The model finds the optimal segmentation of a given chromosome into TADs using a dynamic programming approach that maximizes the sum of TAD-specific probabilistic scores. A hierarchy of domains is derived by merging most similar neighboring domains.

The only user-defined parameter used by PSYCHIC is the size of the window used by DI that we set as described in the DI section.

TADbit (v0.2.51)[20] is suite of tools that include TAD calling. Here, interactions are modeled using Poisson regression and the breakpoint detection algorithm returns the most likely partition according to a penalized Bayesian Information Criterion (BIC).

We did not restrict the maximal size of a TAD (by default, the length of the chromosome) and set to "True" the parameter controlling the search for centromeric region.

3DNetMod (v1.0) [21] is designed for the detection of the hierarchical structure of TADs. The TAD detection problem is defined as a community detection problem in a graph. Precisely, communities are detected by maximizing network modularity by means of a Louvain-like algorithm, that can detect nested structures through variation of the resolution parameter gamma.

A large number of parameters need to be set to run 3DNetMod. The following parameters has been used in our analysis (if provided, recommended values by the authors are indicated in parentheses): region_size=225 (150-300), overlap=100 for bin sizes >= 20kb, 200 for 10kb (idem), logged=True, badregionfilter=True, plateau=3 (3), chaosfilter=False, diagonal_density=0.65 (default 0.95 but should be set to 0.65 in future versions), consecutive_diagonal_zero=20 (default 3, can be relaxed to 20 to capture more regions), num_part=20 (20), pctile_threshold=0 (0), pct_value=0 (0), size_threshold=4 (4), var_thresh1=var_thresh2=var_thresh3=var_thresh4=var_thresh5=100, size_s1 = 400000 (400000), size_s2 = 800000 (800000), size_s3 = 1600000 (1600000), size_s4 = 3000000 (3000000), size_s5 = 12000000 (12000000), boundary_buffer=bin size of data (as suggested by the authors).

HiCExplorer (v1.7.2) [22] Here, the Hi-C matrix is first transformed into a z-score matrix where the z-score of each entry is computed based on the mean and standard deviation of all entries at the same genomic distance. A TAD-separation score is computed for each bin as the mean of the z-scores in a "diamond" submatrix defined according to a given window size. This score is computed for different window sizes, and then averaged for the corresponding bin (low scores are hence indicative of boundaries). For each local minimum below a given threshold, a Wilcoxon rank-sum test is performed to compare the distribution of z-scores within the submatrices centered around the bin and the submatrices upstream and downstream (the lowest of the two p-values is retained). After Bonferroni's correction of the p-values, only those below a given threshold are eventually retained.

For the TAD separation score calculation, we used the minimal possible values for the minimum, maximum, and step of the window size, i.e. 3*binSize, 6*binSize and 1*binSize respectively.
For the boundary filtering, we initially set the minima threshold and p-value cut-off to their default values (both 0.01). Since, with these settings, no domains were identified, we set a p-value threshold of 0.1 following personal communications with the authors.

References

1. Dixon, J. R. *et al.* Topological domains in mammalian genomes identified by analysis of chromatin interactions. *Nature* **485,** 376–380 (2012).

2. Filippova, D., Patro, R., Duggal, G. & Kingsford, C. Identification of alternative topological domains in chromatin. *Algorithms Mol. Biol.* **9,** 14 (2014).

3. Rao, S. S. P. *et al.* A 3D map of the human genome at kilobase resolution reveals principles of chromatin looping. *Cell* **159,** 1665–1680 (2014).

4. Shavit, Y. & Lio', P. Combining a wavelet change point and the Bayes factor for analysing chromosomal interaction data. *Mol. Biosyst.* **10,** 1576–1585 (2014).

5. Lévy-Leduc, C., Delattre, M., Mary-Huard, T. & Robin, S. Two-dimensional segmentation for analyzing Hi-C data. *Bioinforma. Oxf. Engl.* **30,** i386-392 (2014).

6. Wang, Y., Li, Y., Gao, J. & Zhang, M. Q. A novel method to identify topological domains using Hi-C data. *Quant. Biol.* **3,** 81–89 (2015).

7. Crane, E. *et al.* Condensin-Driven Remodeling of X-Chromosome Topology during Dosage Compensation. *Nature* **523,** 240–244 (2015).
8. Malik, L. I. & Patro, R. Rich chromatin structure prediction from Hi-C data. *bioRxiv* 032953 (2015). doi:10.1101/032953
9. Chen, J., Hero, A. O. & Rajapakse, I. Spectral identification of topological domains. *Bioinformatics* **32,** 2151–2158 (2016).
10. Weinreb, C. & Raphael, B. J. Identification of hierarchical chromatin domains. *Bioinformatics* **32,** 1601–1609 (2016).
11. Shin, H. *et al.* TopDom: an efficient and deterministic method for identifying topological domains in genomes. *Nucleic Acids Res.* **44,** e70 (2016).
12. Zhan, Y. *et al.* Reciprocal insulation analysis of Hi-C data shows that TADs represent a functionally but not structurally privileged scale in the hierarchical folding of chromosomes. *Genome Res.* **27,** 479–490 (2017).
13. Oluwadare, O. & Cheng, J. ClusterTAD: an unsupervised machine learning approach to detecting topologically associated domains of chromosomes from Hi-C data. *BMC Bioinformatics* **18,** 480 (2017).
14. Ardakany, A. R. & Lonardi, S. Efficient and Accurate Detection of Topologically Associating Domains from Contact Maps. in *17th International Workshop on Algorithms in Bioinformatics (WABI 2017)* (eds. Schwartz, R. & Reinert, K.) **88,** 22:1–22:11 (Schloss Dagstuhl–Leibniz-Zentrum fuer Informatik, 2017).
15. Yu, W., He, B. & Tan, K. Identifying topologically associating domains and subdomains by Gaussian Mixture model And Proportion test. *Nat. Commun.* **8,** 535 (2017).
16. Wang, X.-T., Cui, W. & Peng, C. HiTAD: detecting the structural and functional hierarchies of topologically associating domains from chromatin interactions. *Nucleic Acids Res.* **45,** e163–e163 (2017).

17. Haddad, N., Vaillant, C. & Jost, D. IC-Finder: inferring robustly the hierarchical organization of chromatin folding. *Nucleic Acids Res.* **45,** e81 (2017).

18. Yan, K.-K., Lou, S. & Gerstein, M. MrTADFinder: A network modularity based approach to identify topologically associating domains in multiple resolutions. *PLOS Comput. Biol.* **13,** e1005647 (2017).

19. Ron, G., Globerson, Y., Moran, D. & Kaplan, T. Promoter-enhancer interactions identified from Hi-C data using probabilistic models and hierarchical topological domains. *Nat. Commun.* **8,** 2237 (2017).

20. Serra, F. *et al.* Automatic analysis and 3D-modelling of Hi-C data using TADbit reveals structural features of the fly chromatin colors. *PLOS Comput. Biol.* **13,** e1005665 (2017).

21. Norton, H. K. *et al.* Detecting hierarchical genome folding with network modularity. *Nat. Methods* **15,** 119–122 (2018).

22. Ramírez, F. *et al.* High-resolution TADs reveal DNA sequences underlying genome organization in flies. *Nat. Commun.* **9,** 189 (2018).
