# Supplementary material for: Comparison of computational methods for the identification of topologically associating domains
Source: Genome Biol. 2018 Dec 10;19:217. doi: 10.1186/s13059-018-1596-9 (PMC6288901; doi:10.1186/s13059-018-1596-9)
Supplement: Supplementary file 2 — Supplementary figures and figures legends. (PDF 359 kb) [file 13059_2018_1596_MOESM2_ESM.pdf]

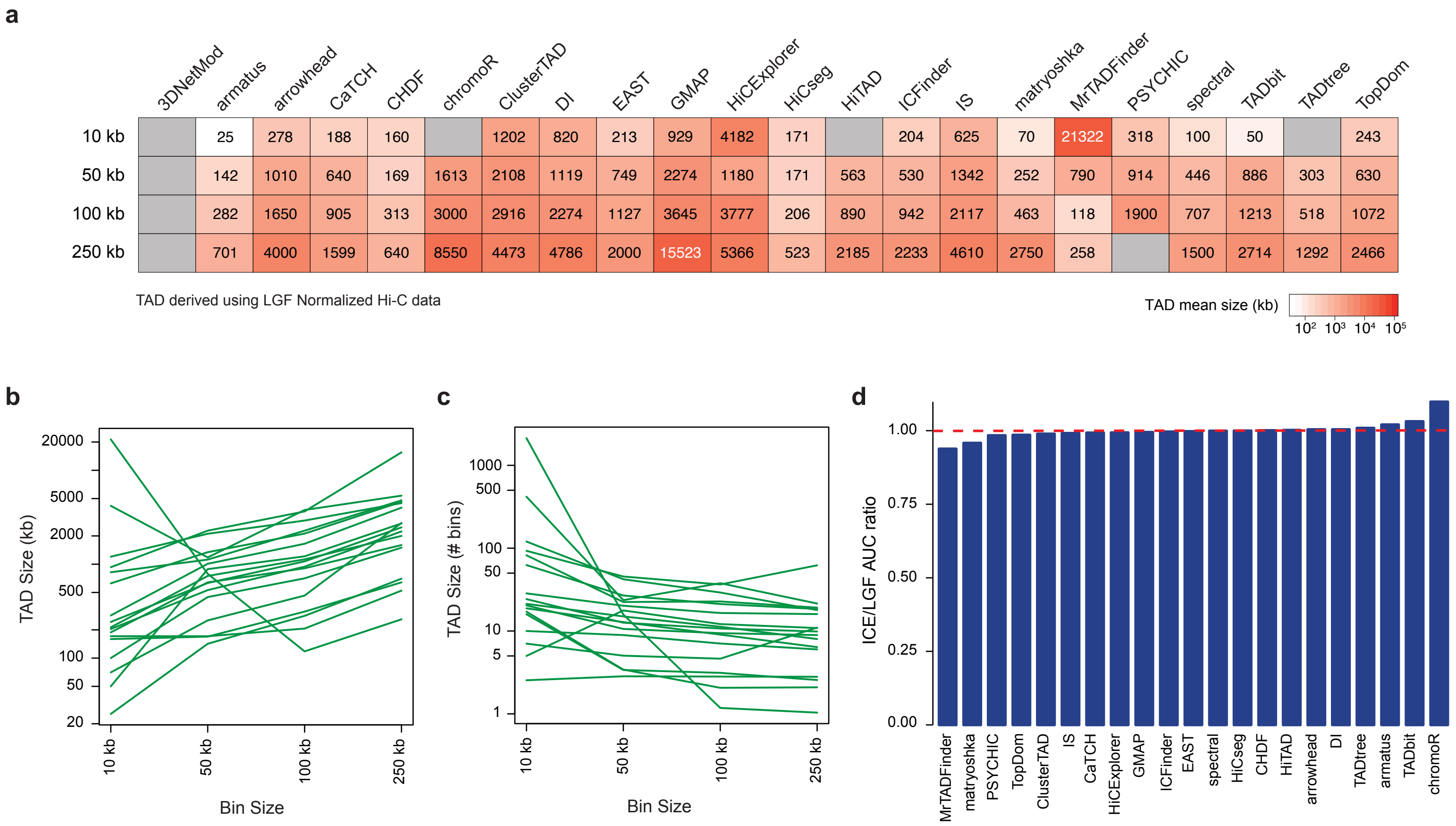

Fig S1

a) Mean size (measured in kb) of the TADs detected in the LGF-normalized Hi-C data of chromosome 4 at four different resolutions (10, 50, 100, 250kb) by each of the 22 TAD callers. Color intensity is proportional to the mean size of the TADs in log-scale, gray boxes correspond to TAD callers that did not successfully identify TADs at a given resolution.

b-c) Variation of the mean size of the TADs measured in kb **(b)** or in number of bins **(c)** across Hi-C matrix resolutions. Each line refers to a TAD caller and only TAD callers that successfully identified TADs at all 4 resolutions are shown.

d) Ratio between the Area Under the Curve (AUC) of the variation of TAD size (measured in kb – panel **b**) across matrix resolutions computed using ICE-normalized Hi-C data and the AUC computed using LGF-normalized Hi-C data.

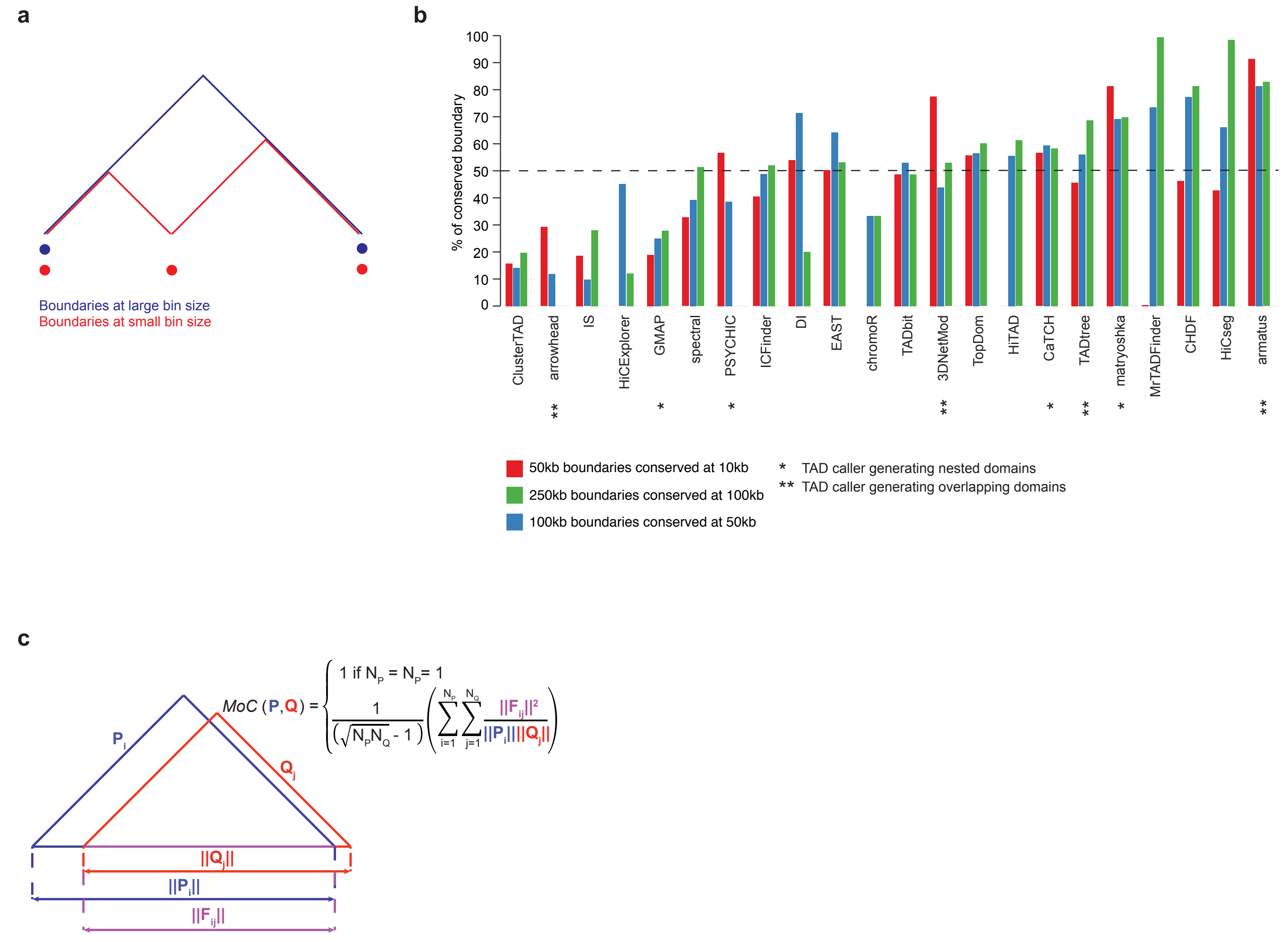

Fig S2

a) Schematic representation of nested domains: two small domains (red) are nested within a large domain (blue).

b) Percentage of boundaries identified from Hi-C matrices at 50, 100, 250kb resolution that contain a boundary identified from Hi-C matrices at, respectively, 10, 50, 100kb resolution. TAD callers are ordered from left to right by increasing mean percentage. TAD callers that return nested TADs are annotated by an asterisk at the bottom.

c) Schematic representation of the calculation of the Measure of Concordance (MoC).

a

Not Available (grey cells)

Number of TADs (colour scale: 1, 10, 10^2, 10^3)

Percentage of reads	3DNetMod	armatus	arrowhead	CaTCH	CHDF	chromoR	ClusterTAD	DI	EAST	GMAP	HiCExplorer	HiCseg	HiTAD	ICFinder	IS	matryoshka	MrTADFinder	PSYCHIC	spectral	TADbit	TADtree	TopDom
0.01	187	778		824	1073	5	66	88	336			1000			117	85	872	168	2115	568	2	140
0.02	248	781		693	1073	10	64	195	300	23		1000			100	70	238	281	2042	2	2	69
0.05	146	793		518	1073	23	70	131	292	31	2	1000			110	187	23	259	1889	167	1	150
0.1	184	768		400	1073	25	72	145	278	63	17	1000		340	109	272	11	235	1730	153	4	216
0.2	194	753		365	1073		72	91	256	89	40	1000		428	103	563	16	167	1159	167	12	263
0.5	284	725	1	323	1072		66	133	245	95	88	1000	92	304	107	535	28	267	746	173	53	286
1	301	726	11	301	1072	34	75	147	227	86	134	1000	276	300	106	449	37	251	513	179	129	296
2	291	715	43	300	1052	22	83	133	215	88	155	1000	280	304	102	539	2254	238	409	187	203	284
5	319	728	75	291	1014	13	76	145	215	71	195	1000	274	319	104	558	3247	226	284	195	223	305
10	321	710	87	283	985		75	133	220	63	206	1000	290	352	104	544	3271	208	233	196	212	292
20	302	711	92	288	960	19	67	131	212	67	228	1000	293	378	104	560	3265	198	186	196	214	303
50	265	708	90	287	1052	13	84	144	208	63	238	1000	284	426	103	547	3234	197	142	193	219	301
100	234	715	94	281	942	13	82	151	205	67	241	1000	293	450	102	544	3207	164	128	187	217	300

b

Not Available (grey cells)

TAD mean size (kb) (colour scale: 10^2, 10^3, 10^4, 10^5)

Percentage of reads	3DNetMod	armatus	arrowhead	CaTCH	CHDF	chromoR	ClusterTAD	DI	EAST	GMAP	HiCExplorer	HiCseg	HiTAD	ICFinder	IS	matryoshka	MrTADFinder	PSYCHIC	spectral	TADbit	TADtree	TopDom
0.01	472	131		207	159	34190	2352	1767	456			171			1100	161	172	1018	81	301	175	1063
0.02	500	138		247	159	17095	2505	807	532	4924		171			1350	241	692	608	84	83950	250	2337
0.05	876	143		330	159	7433	2279	1075	540	4050	51225	171			1195	178	7415	660	90	1024	450	1087
0.1	718	153		427	159	6838	2249	1059	589	2234	9226	171		72	1217	184	15468	727	99	1117	188	754
0.2	646	164		468	159		2262	1563	623	1702	4084	171		221	1305	153	10656	1024	147	1024	508	622
0.5	427	178	3900	529	159		2461	1109	672	1605	1886	171	340	522	1247	173	6089	640	229	988	610	569
1	386	185	886	568	159	5028	1985	1038	727	1768	1236	171	510	539	1263	178	4608	681	333	955	455	549
2	373	188	999	570	163	7773	1884	1141	745	1728	1081	171	578	533	1327	183	75	718	418	914	394	568
5	346	188	1117	588	169	13154	2054	1080	757	1932	859	171	592	506	1298	180	52	757	602	877	365	531
10	341	194	1125	604	174		2088	1179	730	2234	814	171	558	460	1297	187	51	822	734	872	391	554
20	375	195	1045	594	178	9003	2341	1197	761	1901	739	171	555	428	1296	183	51	864	920	873	375	533
50	458	197	1099	596	163	13158	1853	1118	775	2022	712	171	570	380	1314	186	52	868	1205	886	405	538
100	531	195	1072	609	182	13158	1910	1014	792	1899	703	171	552	360	1330	189	52	1043	1336	915	394	539

Fig S3

a-b) Total number **(a)** and mean size **(b)** of TADs detected in the ICE-normalized 50kb-bin Hi-C data of chromosome 6 for increasing sequencing depth (percentage of reads retained from the full list of contacts is indicated by the on the left).

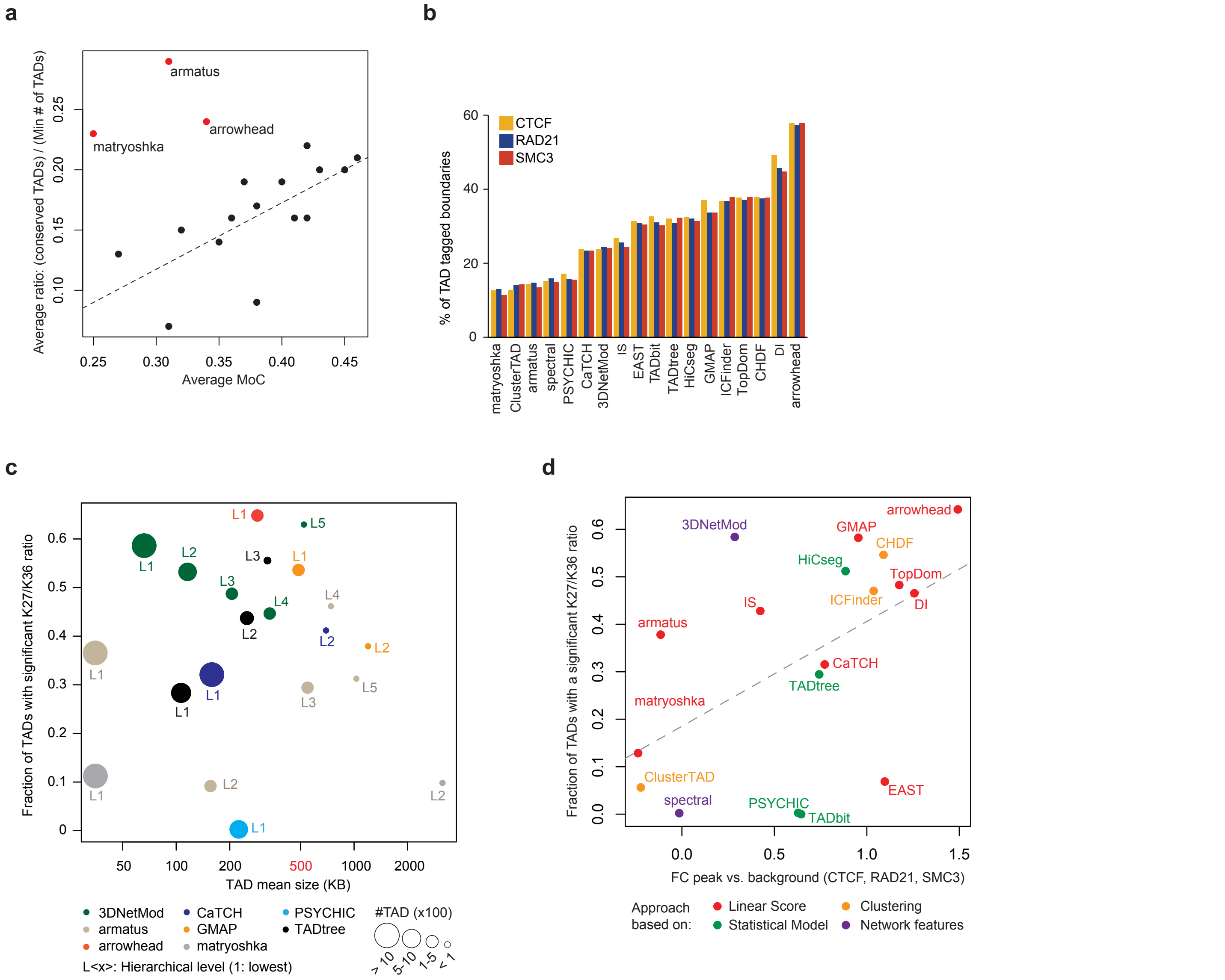

Fig S4

a) For each pair of TAD callers, ratios between number of conserved TADs and the smallest number of TADs identified by the two callers were computed. Average ratios for each TAD caller (Y-axis) are compared to the average Measure of Concordance (MoC) obtained by each TAD caller (X-axis). The dashed line is the linear fit of the values, excluding TAD callers in red (matryoshka, arrowhead, armatus).

b) Percentages of TAD boundaries tagged by CTCF (orange bar), RAD21 (blue bar) or SMC3 (red bar) proteins. TAD callers are ordered from left to right by increasing average percentage of boundaries tagged by the three proteins.

c) Fraction of TADs with significant H3K27me3 / H3K36me3 ratio vs TAD mean size for each hierarchy level of the different callers. Hierarchical levels are labeled by increasing numbers (L1, …, Ln) with L1 being the level including TADs that do not contain nested TAD. The size of the dots is proportional to the number of TADs.

d) Average fold-change (FC) of peak signals of structural proteins (CTCF, RAD21 and SMC3) (X-axis) versus fraction of TADs with significant H3K27me3/H3K36me3 log10-ratio (Y-axis). TAD callers are color coded according to the general approach they adopted. The dashed line indicates the linear fit.

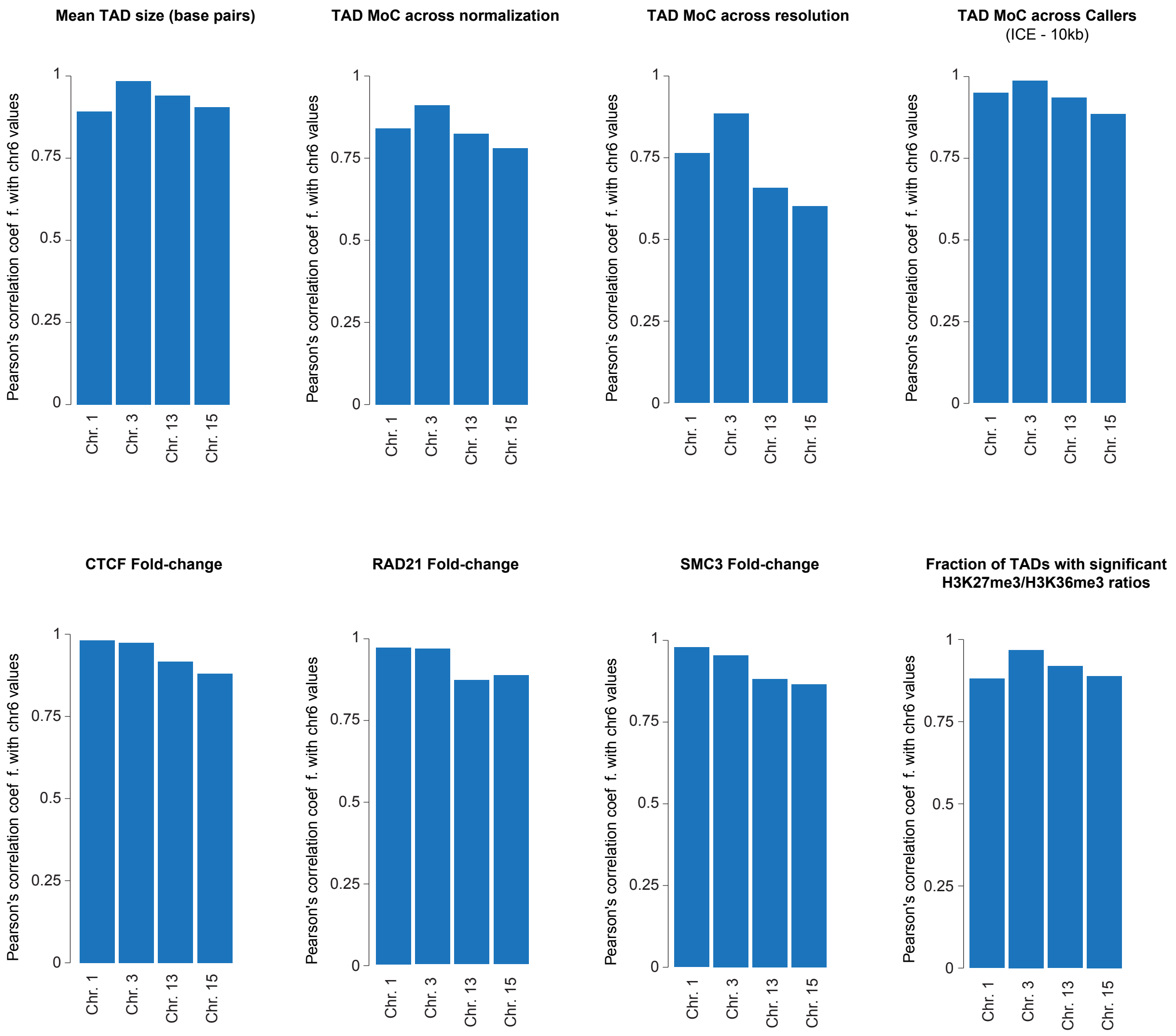

Fig S5

Pearson's correlation coefficients between the results obtained for chromosome 6 and those obtained for chromosomes 1, 3, 13, and 15 for the following analyses (from top left to bottom right): mean TAD size (measured in base pairs), Measure of Concordance (MoC) across normalizations, MoC across resolutions, MoC between TAD callers at 10kb ICE, CTCF fold-change of peak signal, RAD21 fold-change of peak signal, SMC3 fold-change of peak signal, fraction of TADs with significant H3K27me3/H3K36me3 ratios.

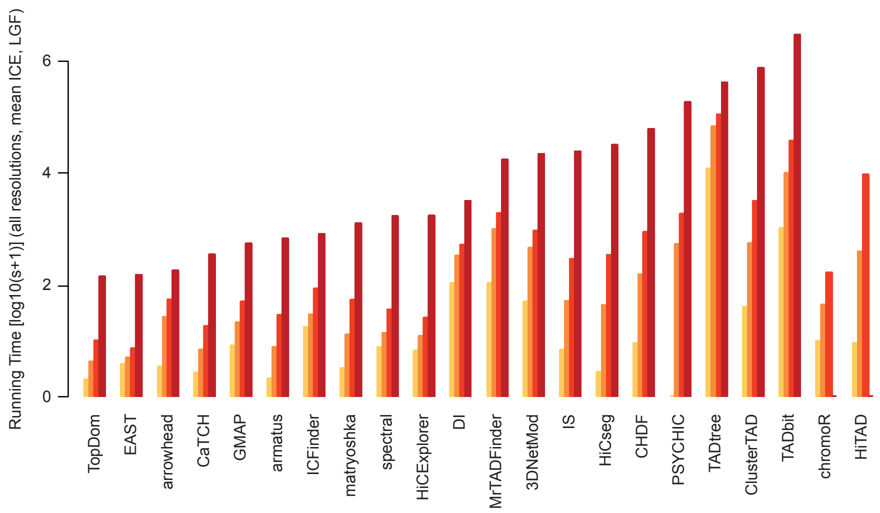

Fig S6

Running time (in log-scale) of the 22 TAD callers (ordered by increasing average value). For each TAD caller, the bars are ordered from left to right by increasing Hi-C data resolution (1000, 250, 100, 50, 10kb) and correspond to the average of the running times obtained for ICE- and LGF-normalized data.
